# Enhanced Method for the Synthesis and Comprehensive Characterization of 1-(4-Phenylquinolin-2-yl)propan-1-one

**DOI:** 10.1021/acsomega.3c04360

**Published:** 2023-11-08

**Authors:** Satheeshkumar Rajendran, Rodrigo Montecinos, Jonathan Cisterna, Kolandaivel Prabha, Karnam Jayarampillai Rajendra Prasad, Sushesh Srivatsa Palakurthi, Alaa A. A Aljabali, Gowhar A. Naikoo, Vijay Mishra, Roberto Acevedo, Koray Sayin, Nitin Bharat Charbe, Murtaza M. Tambuwala

**Affiliations:** † Departamento de Química Orgánica, Facultad de Química y de Farmacia, Pontificia Universidad Católica de Chile, 702843 Santiago de Chile, Chile; ‡ Departamento de Química Física, Facultad de Química y de Farmacia, Pontificia Universidad Católica de Chile, 702843 Santiago de Chile, Chile; § Departamento de Química, Facultad de Ciencias Básicas, Universidad de Antofagasta, Avenida Universidad de Antofagasta 02800, Campus Coloso, Antofagasta 1240000, Chile; ∥ Department of Chemistry, K. S. Rangasamy College of Technology, Tiruchengode 637215, Tamil Nadu, India; ⊥ Department of Chemistry, Bharathiar University, Coimbatore 641046, India; # Department of Pharmaceutical Sciences, Irma Lerma Rangel School of Pharmacy, Texas A&M Health Science Center, Texas A&M University, Kingsville, Texas 78363, United States; ¶ Department of Pharmaceutical Sciences, Faculty of Pharmacy, Yarmouk University, Irbid 566, Jordan; ∇ Department of Mathematics & Sciences, College of Arts & Applied Sciences, Dhofar University, Salalah 211, Oman; ○ School of Pharmaceutical Sciences, Lovely Professional University, Phagwara, Punjab 144411, India; ⧫ Facultad de Ingeniería y Tecnología, Universidad San Sebastián, Bellavista 7, Santiago 8420524, Chile; †† Department of Chemistry, Faculty of Science, Sivas Cumhuriyet University, Sivas 58140, Turkey; ‡‡ Center for Pharmacometrics and Systems Pharmacology, Department of Pharmaceutics, College of Pharmacy, University of Florida, Orlando, Florida 32611, United States; §§ Lincoln Medical School, University of Lincoln, Brayford Pool Campus, Lincoln LN6 7TS, U.K.

## Abstract

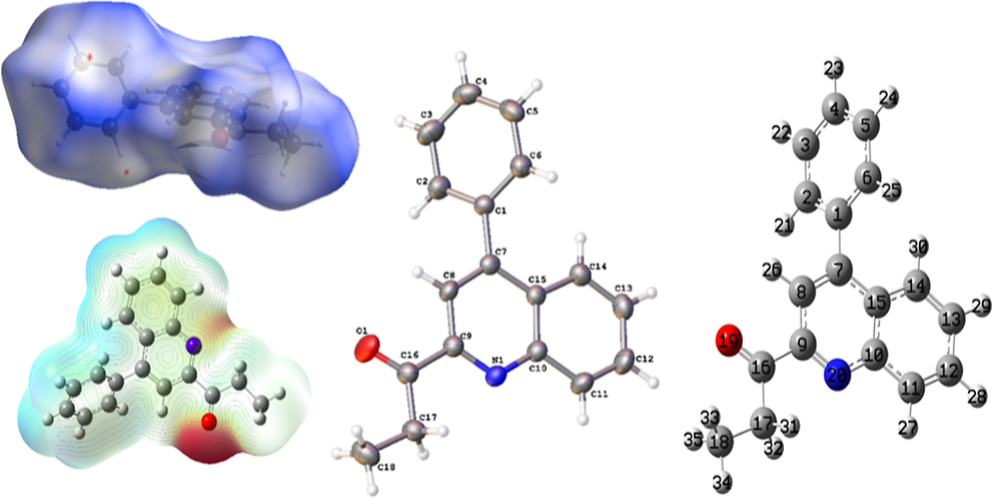

We present an enhanced method for synthesizing a novel compound, 1-(4-phenylquinolin-2-yl)propan-1-one (**3**), through the solvent-free Friedländer quinoline synthesis using poly(phosphoric acid) as an assisting agent. The crystal structure of compound **3** is analyzed using FT-IR, and the chemical shifts of its ^1^H- and ^13^C NMR spectra are measured and calculated using B3LYP/6-311G(d,p), CAM-B3LYP/6-311G(d,p), and M06-2X/6-311G(d,p) basis sets in the gas phase. Additionally, the optimized geometry of quinoline **3** is compared with experimental X-ray diffraction values. Through density functional theory calculations, we explore various aspects of the compound’s properties, including noncovalent interactions, Hirshfeld surface analysis, nonlinear optical properties, thermodynamic properties, molecular electrostatic potential, and frontier molecular orbitals. These investigations reveal chemically active sites within this quinoline derivative that contribute to its chemical reactivity.

## 1 Introduction

The quinoline scaffold is an abundant heterocyclic structural core that exists in numerous naturally found quinoline alkaloids.^1^ The quinoline skeleton is used as a template for several synthetic compounds with various pharmacological properties, including antimalarial, anti-inflammatory, antiasthmatic, antibacterial, antiviral, and antihypertensive and tyrosine kinase inhibiting agents.^1−8^ Researchers continue to seek new methodologies and products for synthesizing quinoline-based moieties. Various classic techniques to synthesize quinoline moiety include the Skraup,^9,10^ Doebner–von Miller,^11^ Pfitzinger,^12^ Conrad–Limpach,^13^ Combes syntheses,^14^ and Povarov reactions.^15,16^ Among them, a notable approach for attaining quinolines and related poly-heterocycles involves Friedländer quinoline synthesis.^17−19^ Among these methods, the Friedländer synthesis is the most effective and prominent protocol for the synthesis of quinolines in recent years.^19−23^ It has become the evergreen synthetic approach to prepare quinoline derivatives by the condensation of easily accessible 2-aminoarylketones with carbonyl compounds possessing a reactive methylene group, followed by cyclodehydration.^24−28^ The most exciting method for improving organic synthesis is continuously reporting new methodologies and catalytic reactions. A solvent-free synthesis is an essential synthetic approach from the perspective of synthetic organic chemistry. Recently, sulfuric acid functionalized heterogeneous catalysts have developed as user-friendly catalysts due to their remarkable advantages over homogeneous catalytic systems, such as prolonged reaction times, enhanced selectivity, the comfort of product purification, ease of operation, low turnover frequency, and surplus amount of catalyst.^29−31^

Based on the above insight, quinoline derivatives were developed by condensing 2-aminoarylketones with carbonyl compounds owning a reactive methylene group, followed by cyclodehydration. Quantum chemical calculations were conducted at the B3LYP/6-311G(d,p), CAM-B3LYP/6-311G(d,p), and M06-2X/6-311G(d,p) levels of theory in the gas phase to optimize the structure, determine vibrational frequencies, chemical shift values using NMR analysis, Hirshfeld surface analysis, highest occupied molecular orbital (HOMO)/lowest unoccupied molecular orbital (LUMO), molecular electrostatic potential (MEP) maps, nonlinear optical (NLO) properties, and thermodynamic properties.^32−37^ Here, we would like to report a convenient and straightforward workup method for the synthesis of quinoline molecule (**3**) using freshly prepared polyphosphoric acid (PPA)^38−41^ (P_2_O_5_ in H_3_PO_4_) under solvent-free conditions from 2-aminobenzophenone (**1**) and pentan-2,3-dione (**2**) through Friedländer quinoline synthesis.

## 2 Instrumentation Methods

### 2.1 FT-IR and NMR Analysis

Fourier transform infrared (FT-IR) BRUKER brand (VECTOR 22) was used to record the FT-IR with spectral data collected in the range 4000–400 cm^–1^. FT-Raman spectrum was recorded using a WiTec Alpha 300 RA Raman-AFM. BRUKER AVANCE III HD-400 [400 MHz (^1^H) and 100 MHz (^13^C)] spectrometers used tetramethyl silane (TMS) as an internal reference for ^1^H NMR and ^13^C NMR. The chemical shifts are observed in parts per million (ppm). Coupling constants (*J*) are reported in hertz (Hz). The terms *J*_o_ and *J*_m_ refer to the ortho coupling constant and meta coupling constant. The terms s, d, t, and dd refer to singlet, doublet, triplet, and doublet of doublet, respectively, and bs refers to a broad singlet. A liquid chromatography–mass spectrometry (LC–MS) experiment was conducted using a UHPLC Eksigent1 coupled with ABSciex1MS detector Triple Quad 4500 model equipment. The sample was injected using a syringe, and the data were acquired in a range of 100.0–600.0 Da, at 200 Da s^–1^ under positive polarity.

### 2.2 Single-Crystal XRD Analysis

The 1-(4-phenylquinolin-2-yl)propan-1-one (**3**) crystals were chosen for measurement. The diffraction data were collected at 296 K on a Bruker D8 Venture diffractometer equipped with a bidimensional CMOS Photon 100 detector. The monochromatic Mo Kα (λ = 0.71073 Å) radiation with a graphite monochromator was used for the data collection. The acquired diffraction frames were integrated using the APEX3 package,^42^ and it was corrected for absorptions with SADABS.^43^ The structure of 1-(4-phenylquinolin-2-yl)propan-1-one (**3**) was solved by intrinsic phasing^44^ using the OLEX2 program.^45^ Then, the obtained structure was refined with full-matrix least-squares methods based on *F*^2^ (SHELXL-2014).^44^ The non-hydrogen atoms were refined with anisotropic displacement parameters during the refinement process of the crystal structure. All hydrogen atoms were included in their calculated positions, assigned fixed isotropic thermal parameters, and confined to riding their parent atoms.

### 2.3 DFT Computational Studies

Molecular structure of the compound in the ground state was optimized, from crystal structure as starting point, using three density functional theory (DFT) methods: B3LYP^46^ the long-range corrected version of B3LYP: CAM-B3LYP^47^ and the Minnesota 06 functional as M06-2X.^48^ All DFT calculations, such as geometrical parameters, energy, IR and NMR spectra, atomic charges, HOMO and LUMO energy, and dipole moment, etc., were performed with the 6-311G(d,p)^49^ basis set level. GaussView 5.0.9^50^ and Gaussian 09AS64L-G09RevD.01^51^ package programs were used to do numerical calculations. ChemBioDraw Ultra Version (13.0.0.3015)^52^ was used as utility program. Energy gap (*E*_gap_) global hardness (η), global softness (*S*), global electronegativity (χ), and chemical potential (μ) were calculated to determine the reactivity of studied compound (**3**) with eqs 1–5,^53−55^ respectively
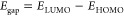
1
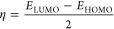
2
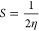
3
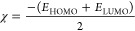
4
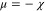
5

### 2.4 Hirshfeld Surface Analyses

Hirshfeld surface analysis^56^ was performed using CrystalExplorer 17.5.^57^ This analysis consists of *d*_norm_ surface plots and 2D (two-dimensional) fingerprint plots.^58^ The mapped electrostatic potentials on the Hirshfeld surfaces using the BLYP/6-31G(d,p) level of theory over a range of ±0.002 au using the TONTO computational package integrated into the program CrystalExplorer.^59^ The analysis was conducted using the crystallographic information file (CIF) of 1-(4-phenylquinolin-2-yl)propan-1-one (**3**) as the input. The normalized contact distance (*d*_norm_) was described in terms of *d*_e_, *d*_i_, and vdW radii of the atoms was calculated using eq 6, where *d*_e_ and *d*_i_ are the lengths from the Hirshfeld isosurface to the nearest external and internal nucleus, respectively, and vdW corresponds to the van der Waals radii of atoms.^60,61^
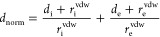
6

## 3 Experimental Details

### 3.1 General

All reagents and chemicals used in the study were purchased from AKSci and Sigma-Aldrich. Unless otherwise indicated, other reagents were obtained from commercial suppliers. Relevant references were given when known compounds were prepared according to the literature procedures. The purity of the synthesized product was demonstrated by TLC silica gel 60 F254 25 aluminum foil 20 × 20 C (purchased from Merck) using petroleum ether and ethyl acetate in the ratio of 95:5 as preparing solvents. Melting points (Mp) were shown on a Kofler Thermogerate apparatus and were uncorrected. They are displayed in degrees of centigrade (°C).

### 3.2 Synthesis

#### 3.2.1 General Procedure for Preparation of 1-(4-Phenylquinolin-2-yl)propan-1-one (**3**)

A mixture of the 2-aminobenzophenone (**1**, 1 mmol) and pentan-2,3-dione (**2**, 1.2 mmol) was heated for 1 h in the presence of freshly prepared PPA reagent (P_2_O_5_ in H_3_PO_4_) as a catalyst without any solvent at 90 °C. The completion of the reaction was analyzed by TLC. The reaction was quenched with an excess amount of saturated sodium carbonate solution (30 mL). Then, the obtained solid was filtered, washed with water, extracted with CH_2_Cl_2_ (3 × 10 mL), and dried over anhydrous sodium sulfate. Evaporation of the solvent was followed by purification via recrystallization from CH_2_Cl_2_ to yield the pure product 2-acetyl-4-arylquinoline (**3**).

White solid; yield 82%, mp 111–112 °C. FT-IR (KBr, cm^–1^) (Figure S1) ν_max_: 3055, 2947, 1697, 1558, 1435, 1373, 1172, 1010.

^1^H NMR (400 MHz, CDCl_3_) (Figure S5) (ppm): δ 1.30 (t, 3H, CH_3_, *J* = 7.20 Hz), 3.46 (q, 2H, CH_2_, *J* = 7.20 Hz), 7.47–7.56 (m, 5H, C_2_-, C_3_-, C_4_-, C_5_-, C_6_-H), 7.59 (t, 1H, C_12_-H, *J* = 8.00 Hz), 7.78 (t, 1H, C_13_-H, *J* = 8.00 Hz), 7.97 (d, 1H, C_14_-H, *J* = 8.00 Hz), 8.08 (s, 1H, C_8_-H), 8.26 (d, 1H, C_11_-H, *J* = 8.00 Hz).

^1^H NMR (400 MHz, DMSO-*d*_6_) (Figure S6) (ppm): δ 1.18 (t, 3H, CH_3_, *J* = 7.20 Hz), 3.41 (q, 2H, CH_2_, *J* = 7.20 Hz), 7.56–7.64 (m, 5H, C_2_-, C_3_-, C_4_-, C_5_-, C_6_-H), 7.75 (t, 1H, C_12_-H, *J* = 8.00 Hz), 7.89–7.95 (m, 3H, C_8_-, C_13_, C_14_-H), 8.26 (d, 1H, C_11_-H, *J* = 8.00 Hz).

^13^C NMR (100 MHz, CDCl_3_) (Figure S10) (ppm): δ 8.14, 30.91, 118.36, 125.85, 128.09, 128.44, 128.58, 128.63, 129.58, 129.73, 130.93, 137.83, 147.83, 149.39, 152.61, 203.29.

^13^C NMR (100 MHz, DMSO-*d*_6_) (Figure S11) (ppm): δ 8.42, 30.72, 118.09, 125.93, 127.73, 129.34, 129.65, 129.84, 130.92, 130.98, 137.52, 147.59, 149.40, 152.64, 202.51. LC–MS for (C_18_H_15_NO) *m*/*z* (%): 261.9 (M + 1, 100).

## 4 Results and Discussion

### 4.1 Synthesis

**Scheme 1:**
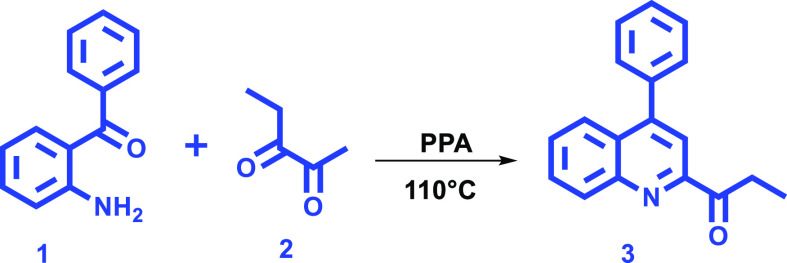
Synthesis of 1-(4-Phenylquinolin-2-yl)propan-1-one (**3**)

The 2-aminobenzophenone (**1**) was treated with pentan-2,3-dione (**2**) in the presence of freshly prepared PPA as a catalyst without any solvent at 90 °C for 1 h to yield 82% of 1-(4-phenylquinolin-2-yl)propan-1-one (**3**) (Scheme 1).

### 4.2 Computational Methods

#### 4.2.1 Full Optimizations

**Figure 1:**
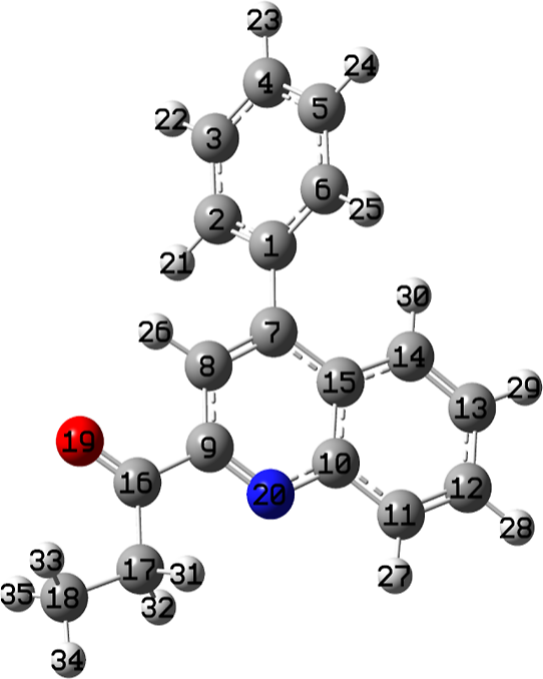
Optimized structure of molecule **3** at the B3LYP/6-311G(d,p) level in vacuum.

The studied compound is fully optimized at each computational level and the optimized structure of molecule **3** is obtained at B3LYP/6-311G(d,p) in the gas phase are given in Figure 1.

#### 4.2.2 FT-IR Analysis

**Figure 2:**
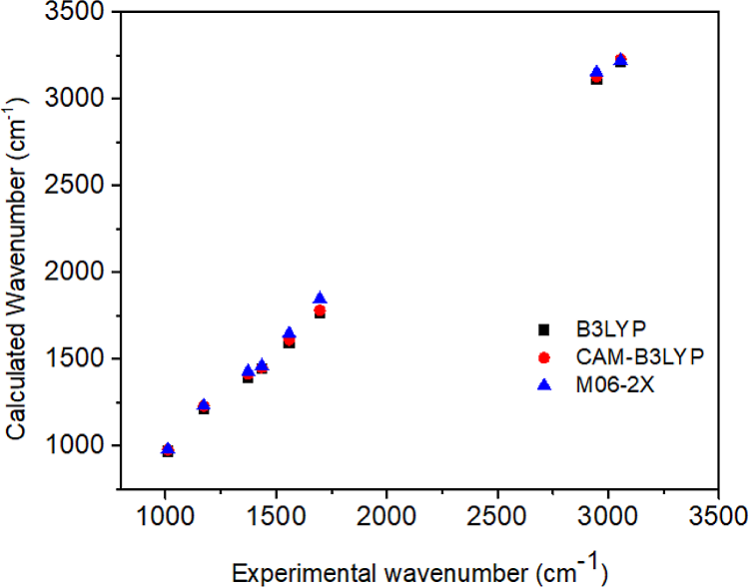
Distribution graphs between experimental and calculated stretching frequencies for molecule **3**.

FT-IR spectrum of molecule **3** is calculated at B3LYP/6-311G(d,p), CAM-B3LYP/6-311G(d,p), and M06-2X/6-311G(d,p) levels of theory, and experimental stretching frequency values are represented as a distribution graph in Figure 2. Furthermore, correlation coefficient for each level. The calculated and experimental stretching frequencies are harmonic frequencies. Some stretching frequencies and correlation coefficients (*R*^2^) are shown in Table 1.

**Table 1:** FT-IR Frequencies (cm^–1^) (Figures S1–S4)

assignments	experimental	B3LYP	CAM-B3LYP	M06-2X
ν_CH asymmetric_	3055.24	3209.2–3163.7	3227.9–3182.1	3219.3–3170.3
ν_CH symmetric_	2947.23	3108.9–3104.4	3128.0–3123.2	3150.8–3139.7
–C=O	1697.36	1764.6	1779.9	1844.9
–C=N	1558.48	1592.2	1610.7	1646.0
=C–H	1435.04	1443.1	1450.2	1458.7
H–C–H	1373.20	1392.9	1412.3	1427.2
N–C–C=O	1172.72	1215.3	1228.6	1230.4
Ph ring	1010.70	967.5	975.4	978
*R*^2^		0.9849	0.9838	0.9715

The B3LYP/6-311G(d,p) level is the best calculation level in Table 1 and Figure 2 due to the fact that the highest *R*^2^ is obtained in this level. After those computational results in the B3LYP/6-311G(d,p) level are given for other computational parts. In the FT-IR (KBr, cm^–1^) spectrum (Figure S1) of molecule **3**, the C=O stretching at 1697 cm^–1^ and C=N stretching at 1558 cm^–1^ were found. The theoretical values of FT-IR, which are given by B3LYP/6-311G(d,p) (Figure S2), CAM-B3LYP/6-311G(d,p) (Figure S3), and M06-2X/6-311G(d,p) (Figure S4) levels of theory are mostly in good agreement, as shown in Table 1.

#### 4.2.3 FT-NMR Analysis

The ^1^H NMR (400 MHz) and ^13^C NMR (100 MHz) spectra of molecule **3** were recorded in CDC_l3_ and DMSO-*d*_6_ with TMS as the internal standard. Simulated ^1^H and ^13^C NMR spectra were calculated for each level. Experimental and calculated results in the B3LYP/6-311G(d,p) level for molecule **3** are given in Tables 2 and 3. For other computational results, they are given in Tables S1 and S2. Within ^1^H NMR by using CDCl_3_ (Figure S5) or DMSO-*d*_6_ (Figure S6) solvents, aliphatic proton peaks appeared in the upfield region between δ 1.18 and 3.46. The aromatic protons peaks were observed in the downfield region at δ 7.47–8.26. The chemical shift of the C_12_ proton DMSO-*d*_6_ is in the range of δ 7.75; however, in CDCl_3_, it is at about δ 7.59. The C_13_ proton in DMSO-*d*_6_ as a multiplet appeared in the range of δ 7.89–7.95 but in CDCl_3_ at δ 7.78. The C_18_ aliphatic proton showed in CDCl_3_ at δ 1.30 but in DMSO-*d*_6_ at δ 1.18 due to the solvent polarity. The ^13^C NMR spectrum shows the presence of 18 carbons. The characteristic signals at δ 203.29 and δ 202.51 were due to −C_16_=O using CDCl_3_ (Figure S10) and DMSO-*d*_6_ (Figure S11) solvents, respectively. The characteristic signals in the aliphatic region at δ 8.14 (C_18_), 30.91 (C_17_) and δ 8.42 (C_18_), 30.72 (C_17_) appeared using CDCl_3_ (Figure S10) and DMSO-*d*_6_ (Figure S11) solvents, respectively. All other aromatic carbons appeared in the region of δ 118.09–152.64. From DEPT-135 spectra, 30.91 (C_17_) and 30.72 (C_17_) were shown in CDCl_3_ (Figure S15) and DMSO-*d*_6_ (Figure S16) solvents, respectively, which is identified in the downfield region due to the even number of protons.

**Table 2:** ^1^H-NMR Chemical Shift δ (ppm) (Figures S5–S9)

atoms	CDCl_3_	DMSO-*d*_6_	B3LYP/6-311G(d,p)
C2(H)	7.47–7.56	7.56–7.64	7.56
C3(H)			7.51
C4(H)			7.52
C5(H)			7.64
C6(H)			7.66
C8(H)	8.08	7.89–7.95	8.3
C11(H)	8.26	8.26	8.38
C12(H)	7.59	7.75	7.79
C13(H)	7.78	7.89–7.95	7.6
C14(H)	7.97	7.89–7.95	8.14
C17(H2)	3.46	3.41	3.41
C18(H3)	1.30	1.18	1.19

**Table 3:** ^13^C-NMR Chemical Shift δ (ppm) (Figures S10–S14)

atoms	CDCl_3_	DMSO-*d*_6_	B3LYP/6-311G(d,p)
C1	137.83	137.52	160.92
C2	129.58	129.34	150.82
C3	128.44	129.65	147.11
C4	128.58	129.65	148.21
C5	128.58	129.65	149.43
C6	129.58	129.34	149.69
C7	149.39	149.40	171.32
C8	118.36	118.09	137.32
C9	152.61	152.64	171.25
C10	147.83	147.59	167.82
C11	130.93	130.92	152.63
C12	129.73	130.98	148.56
C13	128.63	129.84	147.72
C14	128.09	127.73	145.88
C15	125.85	125.93	147.44
C16	203.29	202.51	220.35
C17	30.91	30.72	50.15
C18	8.14	8.42	24.14

**Figure 3:**
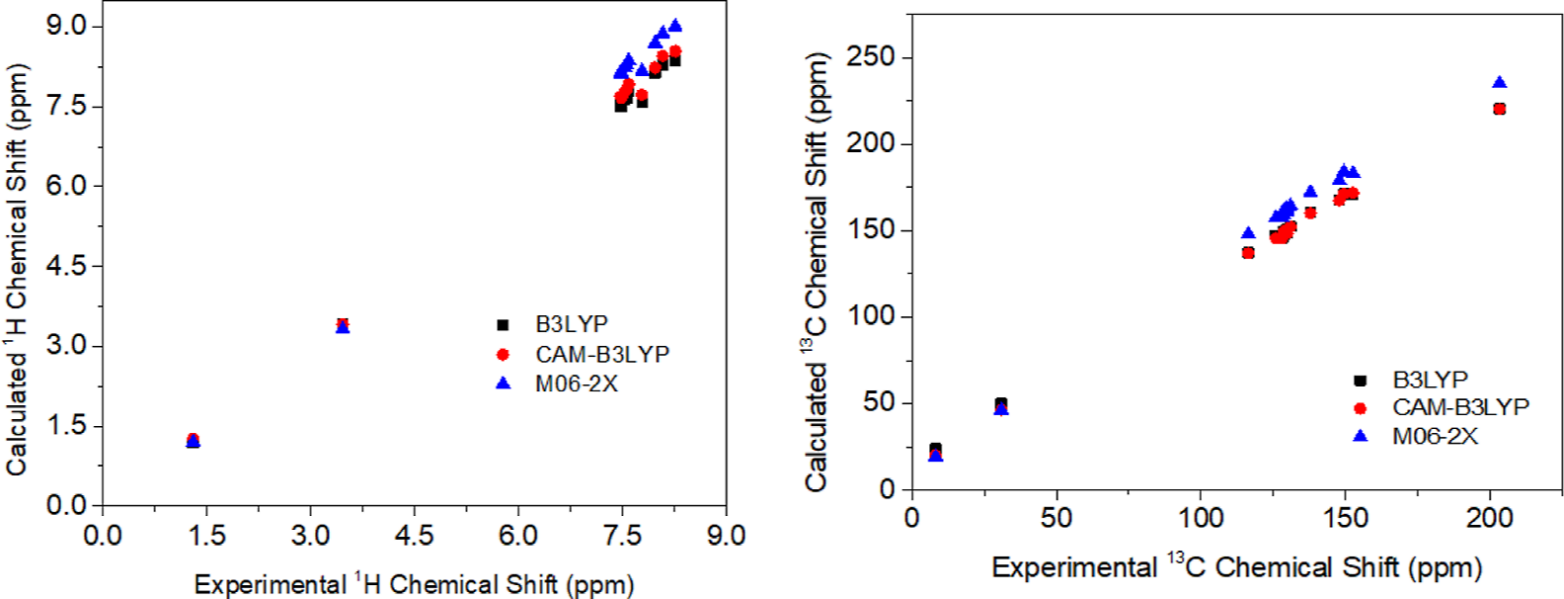
Distribution graphs between calculated and experimental ^1^H and ^13^C NMR chemical shifts for molecule **3**.

The ^1^H and ^13^C NMR spectra of molecule **3** were calculated by using B3LYP/6-311G(d,p), CAM-B3LYP/6-311G(d,p), and M06-2X/6-311G(d,p) levels of density functional calculations, and experimental chemical shift values are represented as a distribution graph in Figure 3. The theoretical ^1^H and ^13^C chemical shift values compared with experimental ^1^H and ^13^C chemical shift values showed in Tables 2 and 3. The theoretical ^1^H and ^13^C chemical shift results for (**3**) are mostly closer to the experimental ^1^H and ^13^C shift data. The optimized electronic structure (Figure 1), FT-IR (Figures S2–S4), and NMR (Figures S7–S9 and S12–S14) spectroscopy of molecule **3** were performed using at B3LYP/6-311G(d,p), CAM-B3LYP/6-311G(d,p), and M06-2X/6-311G(d,p) levels of theory. The absence of imaginary frequencies for the optimized geometry of molecule **3** suggests a suitable conversion of the model calculations.

#### 4.2.4 Single-Crystal XRD Studies

##### 4.2.4.1 Crystal and Molecular Structure

**Figure 4:**
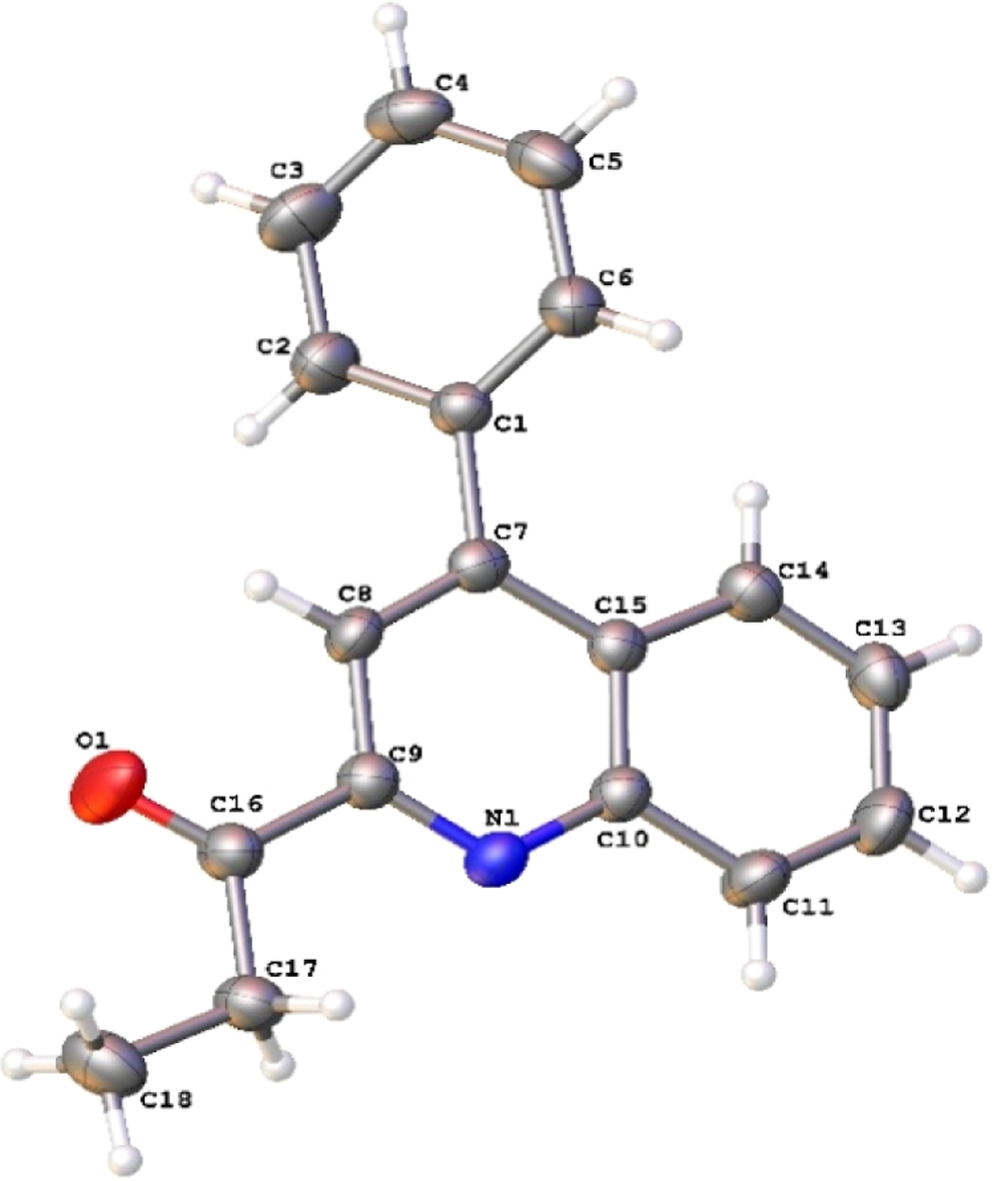
ORTEP plot for molecule **3**. Thermal ellipsoids were drawn with 50% of probability.

The molecular structure of 1-(4-phenylquinolin-2-yl)propan-1-one (**3**) crystallizes in a orthorhombic system, in the space group *P*2_1_2_1_2_1_ (*Z* = 4), with normal bond angles and distances^62^ and similar to related compounds.^63,64^ The fragments linked to the quinoline ring are practically planar, except for phenyl ring attached to quinoline ring, were their mean planes have a torsion angle of 49.5(1)°, in agreement with 56.75(8) and 61.35(6) found in other similar compounds^63,64^ (see Figure 4 for more details). A summary of the details about crystal data, collection parameters, and refinement is included in Table 4, and additional crystallographic details are included in the CIF files. ORTEP (Figure 4) views were drawn using OLEX2 software.^45^

**Table 4:** Crystal Data Parameters for Molecule **3**

empirical formula	C_18_H_15_NO
CCDC number	2077085
formula mass, g mol^–^^1^	261.31
collection *T*, K	296.86
crystal system	Orthorhombic
space group	*P*2_1_2_1_2_1_
*a* (Å)	7.2696(6)
*b* (Å)	10.6810(8)
*c* (Å)	17.8044(14)
*V* (Å^3^)	1382.45(19)
*Z*	4
ρ_calcd_ (g cm^–3^)	1.255
crystal size (mm)	0.25 × 0.23 × 0.19
*F*(000)	552.0
abs coeff (mm^–1^)	0.078
2θ range (deg)	5.958 to 56.534
range *h*, *k*, *l*	–9/9, −14/14, −23/23
no. total refl.	26,163
no. unique refl.	3433 [*R*int = 0.0836, *R*sigma = 0.0468]
comp. θ_max_ (%)	99.9
max/min transmission	0.983/0.980
data/restraints/parameters	3433/0/183
final *R* [*I* > 2σ(*I*)]	*R*1 = 0.0526, wR2 = 0.0980
*R* indices (all data)	*R*1 = 0.0976, wR2 = 0.1162
goodness of fit/*F*^2^	1.022
largest diff. peak/hole (eÅ^–3^)	0.23/–0.15
Flack parameter	–0.7(10)

The calculated geometrical parameters (bond length, bond angle, and dihedral angle) of molecule **3** and single-crystal X-ray diffraction (XRD) data are listed in Table 5, following the atom numbering scheme given in Figure 1.

**Table 5:** Optimized Geometrical Parameters (Bond Lengths, Bond Angles, and Dihedral Angels) of Molecule **3** Using B3LYP/6-311G(d,p), CAM-B3LYP/6-311G(d,p), and M06-2X/6-311G(d,p) Levels of Theory in Comparison with Experimental XRD Studies

parameters	bond length (A)		parameters	bond angles (deg)		parameters	dihedral (deg)	
	XRD	B3LYP/6-311G(d,p)		XRD	B3LYP/6-311G(d,p)		XRD	B3LYP/6-311G(d,p)
C1–C2	1.39	1.39	C1–C2–C3	120.8	120.61	C2–C1–C7–C8	–47.23	–55.86
C2–C3	1.39	1.39	C2–C3–C4	120.29	120.15	C6–C1–C7–C15	132.91	121.9
C3–C4	1.37	1.39	C3–C4–C5	119.73	119.69	C7–C15–C14–C13	–178.86	–179.78
C4–C5	1.37	1.39	C4–C5–C6	120.59	120.2	C7–C8–C9–N1	–0.97	0.1
C5–C6	1.38	1.39	C5–C6–C1	120.08	120.54	C9–N1–C10–C11	–177.84	–178.85
C1–C7	1.49	1.49	C6–C1–C7	122.14	121.08	C9–N1–C10–C15	1.29	0.587
C7–C8	1.37	1.37	C1–C7–C8	118.58	118.82	C9–C16–C17–C18	179.8	179.85
C7–C15	1.42	1.43	C1–C7–C15	123.56	122.34	N1–C9–C16–C17	4.27	0.369
C8–C9	1.41	1.41	C7–C8–C9	120.52	119.9	N1–C10–C11–C12	178.71	178.52
C9–N1	1.32	1.31	C7–C15–C14	124.25	123.88			
N1–C10	1.37	1.36	C8–C9–N1	123.52	123.76			
C10–C11	1.41	1.42	C8–C9–C16	118.7	118.55			
C10–C15	1.43	1.42	C9–N1–C10	117.4	118.01			
C11–C12	1.36	1.36	N1–C10–C11	117.73	117.74			
C12–C13	1.4	1.41	N1–C10–C15	123.03	122.73			
C13–C14	1.36	1.37	C10–C11–C12	120.52	120.58			
C14–C15	1.42	1.42	C10–C15–C14	118.12	118.37			
C9–C16	1.51	1.51	C11–C12–C13	120.48	120.08			
C16–O1	1.21	1.21	C12–C13–C14	120.61	120.66			
C16–C17	1.49	1.51	C13–C14–C15	121.03	120.756			
C17–C18	1.51	1.52	C9–C16–C17	118.37	117.73			
			C9–C16–O1	119.4	119.67			
			C16–C17–C18	113.8	112.87			

**Figure 5:**
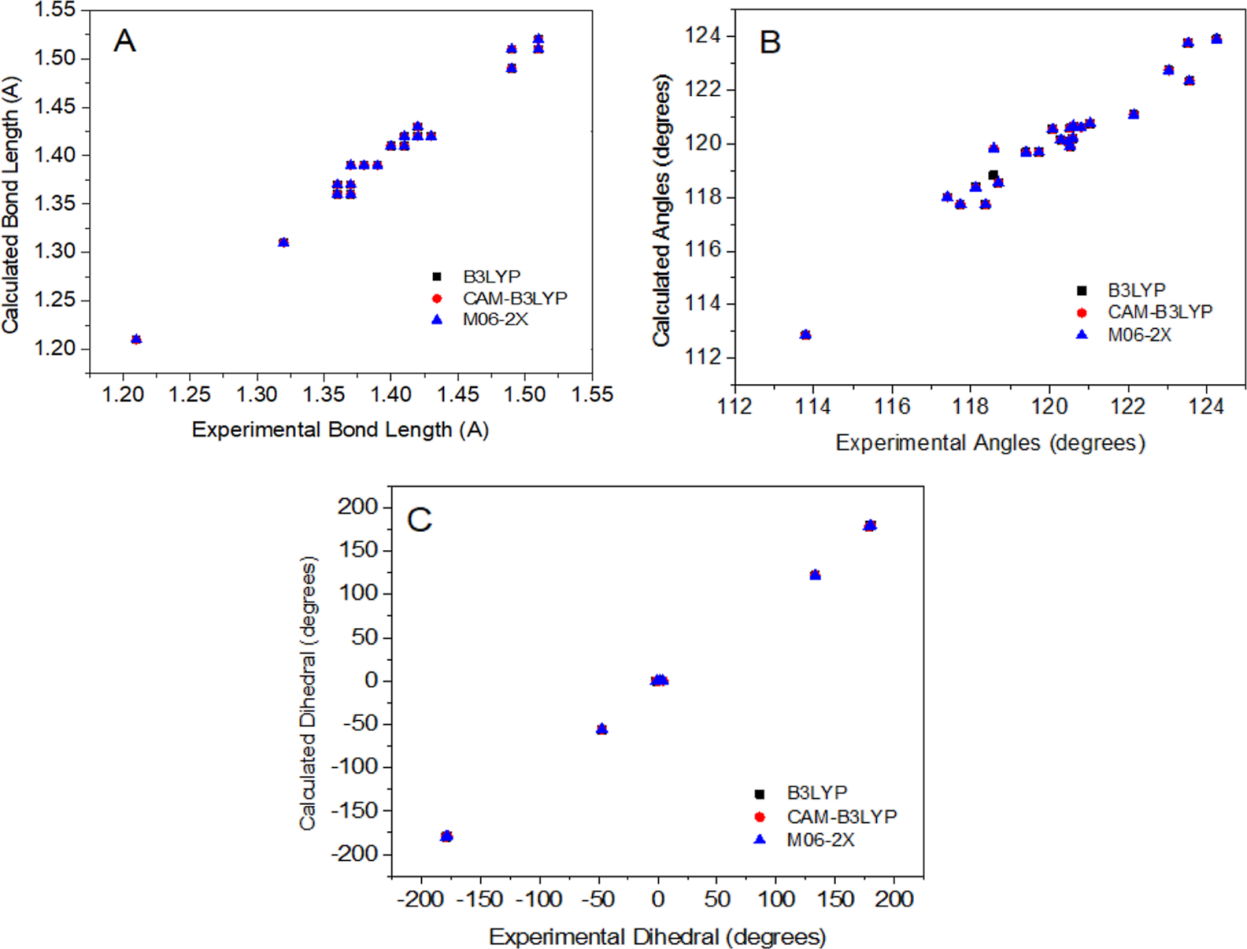
Correlation of experimental and calculated structural parameters for molecule **3**. (A) Bond length, (B) bond angles, and (C) dihedral angles.

The distribution graphs show the agreement between calculated and experimental structural parameters. Correlation coefficients (*R*^2^) are 0.9827, 0.9794, and 0.9973 for bond length, bond angles, and dihedral, respectively, in Figure 5. According to Table 5 and Figure 5, the calculated and experimental values of geometrical parameters were satisfactory.

#### 4.2.5 Noncovalent Interactions

**Figure 6:**
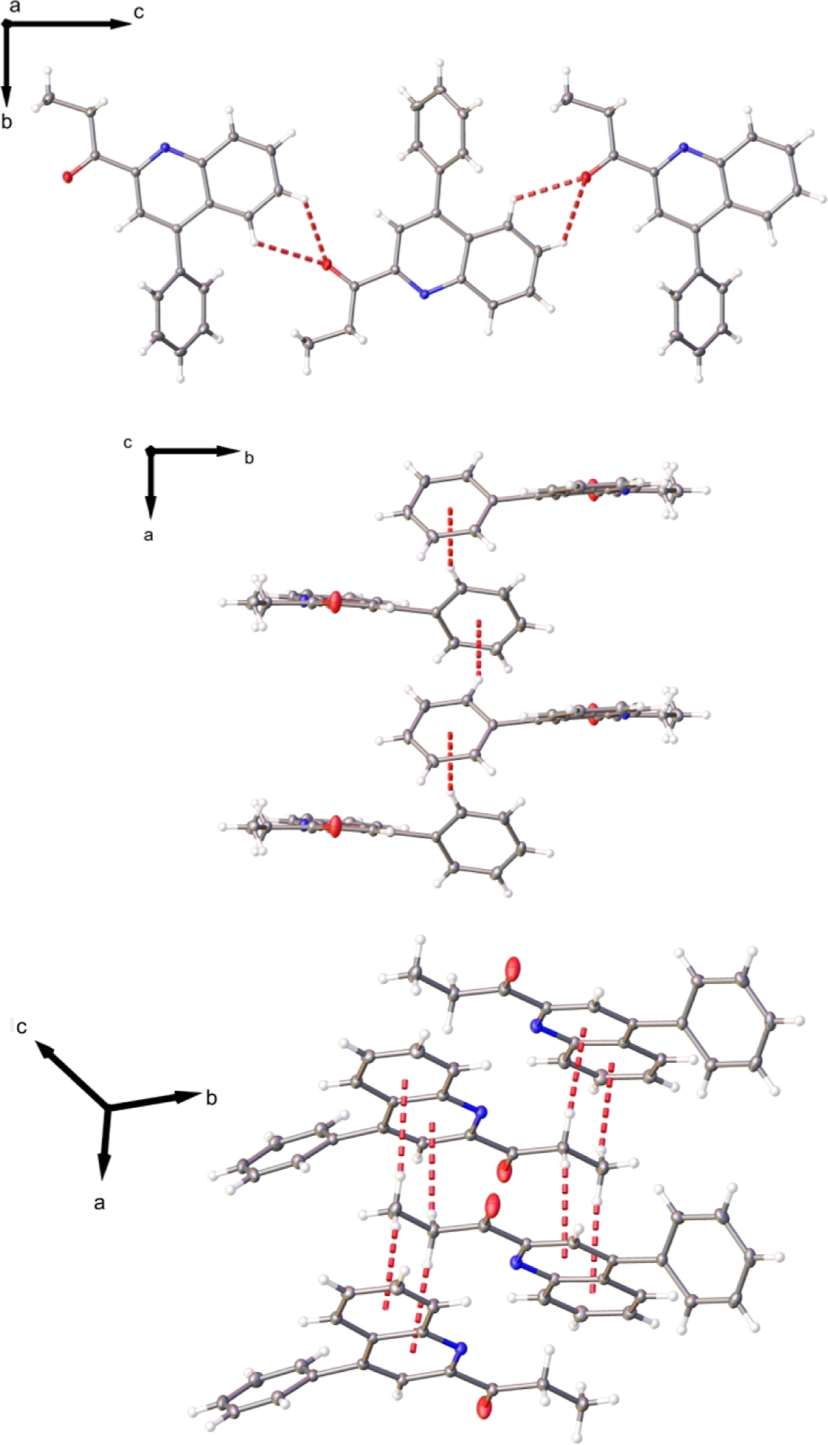
Crystal packing of molecule **3**.

Additionally, the crystal structure does not show classic hydrogen bonds between neighbor molecules. However, one C–H···O intermolecular bifurcated hydrogen bond interaction is observed with graph-set notation **C**_**1**_^**1**^(**8**), **C**_**1**_^**1**^(**9**), and **R**_**1**_^**2**^(**5**).^65^ Large C–H···π interactions are also observed in the crystal structure of the title compound. Both interactions observed in the crystal structure lie in each screw axis present in the space group. Moreover, the distances between involved atoms is larger than the sum of the VdW radii^60,61^ (see Figure 6 and Table 6 for more details).

**Table 6:** Hydrogen Bond Interactions for Molecule **3**

atoms	D–H	H···A	D···A	D–H···A
C13–H13···O1^b^	0.93	2.732	3.314(4)	121.5
C14–H14···O1^b^	0.93	2.829	3.366(4)	117.8
C2–H2···CNT1^c^^,^^a^	0.93	2.739	3.611(3)	159.4
C17–H17B···CNT2^d^^,^^a^	0.97	2.841	3.639(4)	146.7
C18–H18C···CNT3^d^^,^^a^	0.93	2.739	3.611(3)	159.4

a CNT = centroid; 1: C1/C2/C3/C4/C5/C6; 2: C7/C8/C9/N1/C10/C15; 3: C10/C11/C12/C13/C14/C15.

b 3/2 – *x*, 1 – *y*, −1/2 – *z*.

c –1/2 + *x*, 1/2 – *y*, 1 – *z*.

d –1/2 + *x*, 3/2 – *y*, 1 – *z*.

#### 4.2.6 Hirshfeld Surface Analyses

**Figure 7:**
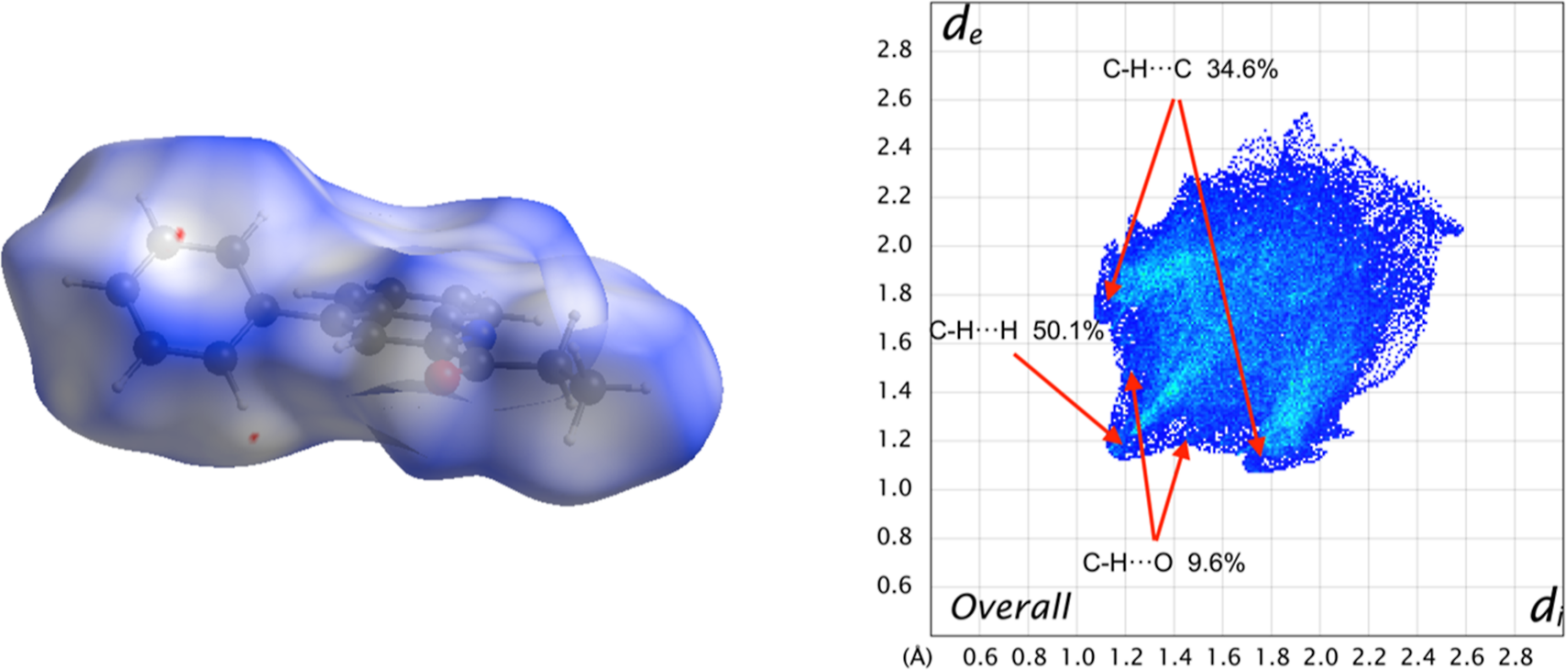
*d*_norm_ surface for molecule **3** (left) and its overall 2D fingerprint plot (right), indicating the most important contacts in the crystal packing. Red and blue colors indicate strong and weak interactions, respectively. Isovalues range from −0.0051 (red) to +1.3934 (blue).

Hirshfeld surface analysis was utilized as a complementary analysis for the crystal packing contacts. The intermolecular interactions are mainly constituted by C–H···O, which are shown as red (*d*_norm_ < VdW radii), white (*d*_norm_ = VdW radii), and blue (*d*_norm_ > VdW radius) spots in the *d*_norm_ surfaces for all compounds. Furthermore, there is evidence of some other interesting weak contacts with less impact on the crystal structure, such as C–H···π contacts. The reciprocal contacts and their corresponding contributions are displayed in Figure 7 and all fingerprint plots with *d*_norm_ (where *d*_norm_ = *d*_i_ + *d*_e_) surfaces for their intermolecular contacts were calculated at the BLYP/6-31G(d,p) level of theory in the cluster of radius 3.8 Å around the molecule.^57^

The reciprocal contacts for C–H···O appear as a wide symmetrical wing with *d*_e_ + *d*_i_ ∼ 2.7, corresponding to classical hydrogen bond interactions. However, this is larger than the sum of the VdW radii, as mentioned above. In the case of C–H···N interactions, the distance is slightly higher than the VdW radii for N and H atoms (*d*_e_ + *d*_i_ > 2.75 Å),^61^ where the contribution is negligible. Remarkably, The interatomic contacts of C–H···H interactions display a *d*_i_ + *d*_e_ ∼ 2.4 Å = 2.4 Å, with a contribution of around 50.1%, creating a nonsignificant effect over molecular packing in the crystal structure stabilization, in other words, these contacts are slightly larger than the sum of the VdW radii for these atoms.^60,61^

In the Hirshfeld surface analysis, there is another type of weak interactions. For example, the contribution of H···π type interaction is around 34.6%, when *d*_e_ + *d*_i_ of ∼2.9 Å. This was analyzed with a shape index, allowing us to determine the presence of these weak interactions. The yellow-orange spots exhibit surface subsidence due to the proximity of the neighboring moieties and the blue-green spots demonstrate the reciprocal contacts of the moieties that generate the subsidence.

**Figure 8:**
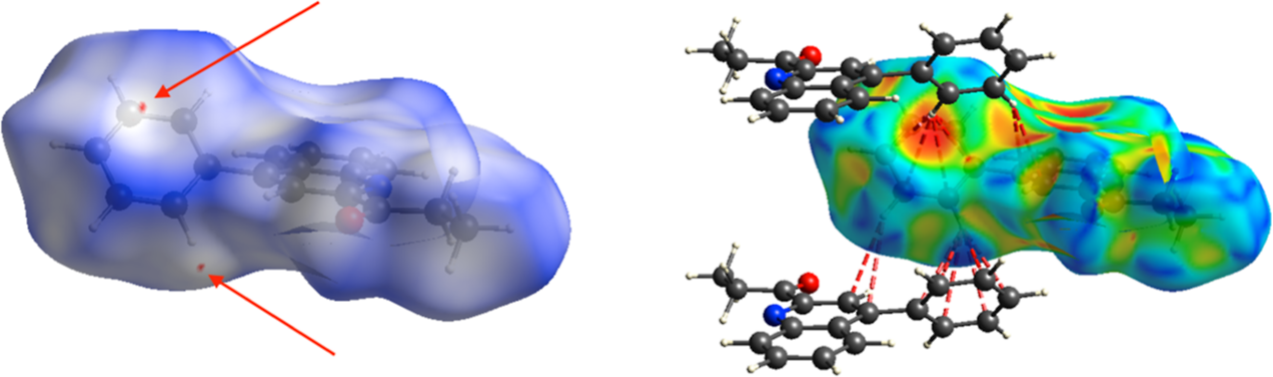
*d*_norm_ surface for molecule **3** (left) and its shape index plot (right) indicate the C–H···π contacts in the crystal packing. Isovalues range from −0.0051 (red/orange) to +1.3934 (blue).

In this context, their corresponding counterparts, where the interaction between hydrogens and delocalized π-clouds bonds are demonstrated when constructing the crystal structure, are shown in Figure 8. However, the sum of VdW radii for C and H atoms interacting between them is slightly larger than their theoretical value (2.95 vs 2.9 Å).^60,61^ Thus, these types of interactions can be reflected weakly as for the *d*_norm_ surface.

#### 4.2.7 Frontier Molecular Orbitals, MEP Maps, and MEP Contours

**Figure 9:**
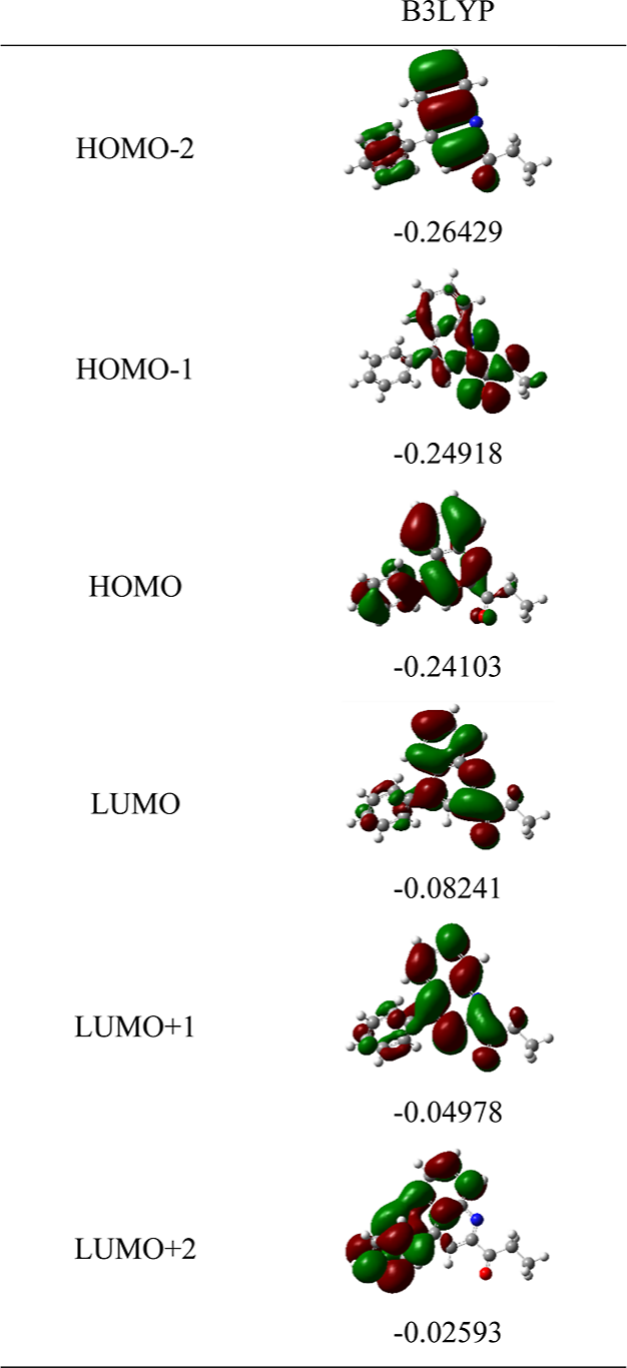
Molecular orbital (MO) energy diagrams (eV) from HOMO – 2 to LUMO + 2 at B3LYP/6-311G(d,p) molecule **3** in the gas phase.

**Figure 10:**
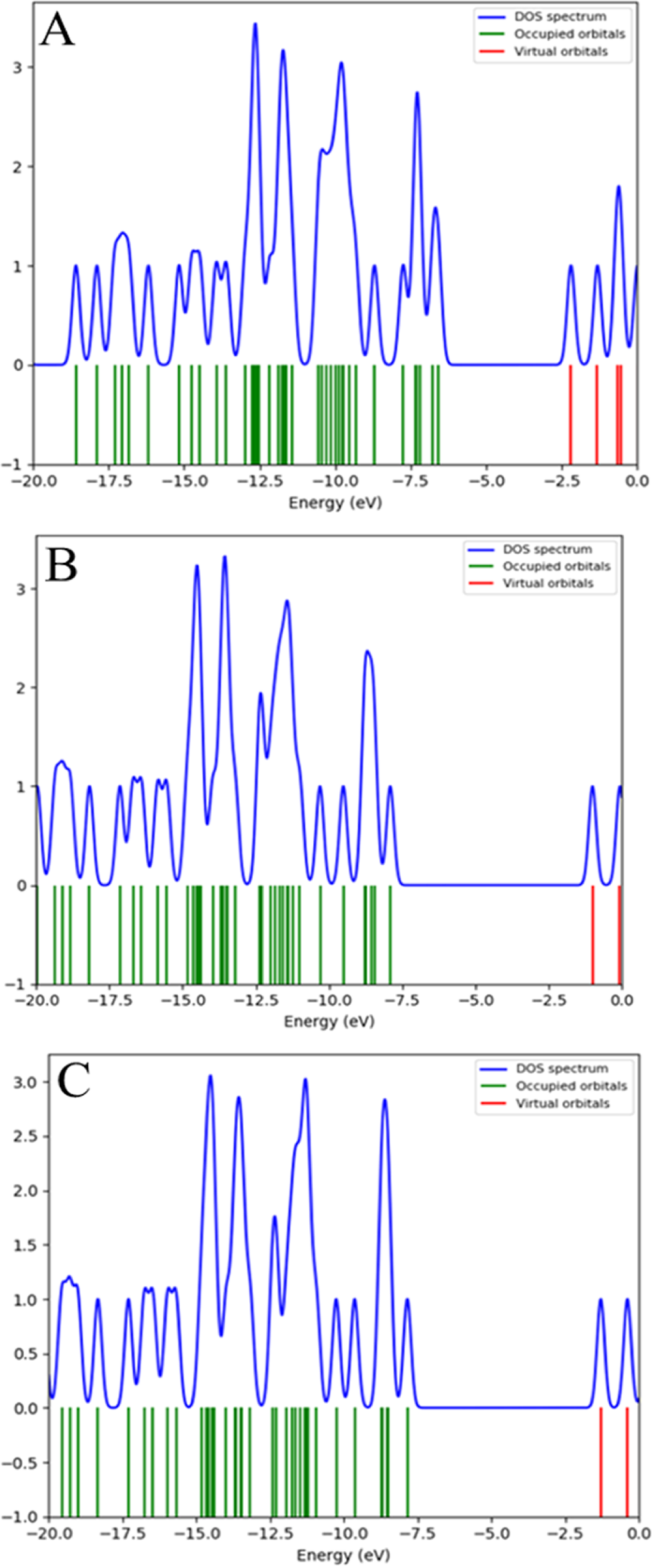
Simulated density of states spectrum of molecule **3** with (A) B3LYP/6-311G(d,p), (B) CAM-B3LYP/6-311G(d,p), and (C) M06-2X/6-311G(d,p) levels of theory.

The DFT calculations of MOs and their energy diagrams were executed at the B3LYP/6-311G(d,p), CAM-B3LYP/6-311G(d,p), and M06-2X/6-311G(d,p) levels of theory in the gas phase. Frontier molecular orbitals (FMOs), such as the HOMO and the LUMO, were exploited to determine the chemical reactivity. The distributions of the energy diagrams from HOMO – 2 to LUMO + 2 for molecule **3** are given in Figure 9 at the B3LYP/6-311G(d,p) level. The results at other calculation levels are listed in Figure S19 (Supporting Information). It is noticed that electrons in HOMO are mostly localized on the quinoline ring of molecule **3**. The presence of electrons in LUMO is also located in the same region of the quinoline ring, indicating the presence of increased chemical activity. The values of hardness, softness, chemical potential, and electronegativity for molecule **3** in the gas phase with B3LYP/6-311G(d,p), CAM-B3LYP/6-311G(d,p), and M06-2X/6-311G(d,p) levels of theory basis sets. In this report, the character of the MOs is calculated using the same levels of theory, and the density of the states (DOS) is shown in Figure 10. DOS plot exhibits the MO compositions at the different energy levels of molecule **3**. Moreover, the DOS is also notable for numerous calculations, such as estimating the occupancy of states and virtual orbitals of molecule **3** using red and green lines in the spectrum.^66,67^ The mentioned quantum chemical parameters are calculated using eqs 1–5 and are given in Table 7.

**Table 7:** FMO Energies, Chemical Potential (μ), Hardness (η) Softness (*S*), and Electronegativity (χ) Parameters for the Molecule **3** Calculated with B3LYP/6-311G(d,p), CAM-B3LYP/6-311G(d,p), and M06-2X/6-311G(d,p) Levels of Theory

property	B3LYP/6-311G(d,p)	CAM-B3LYP/6-311G(d,p)	M06-2X/6-311G(d,p)
*E*_HOMO_	–0.24103	–0.29054	–0.28706
*E*_LUMO_	–0.08241	–0.03665	–0.04792
*E*_GAP_	0.15862	0.25389	0.23914
chemical potential (μ)	–0.16172	–0.16359	–0.16749
global hardness (η)	0.07931	0.12695	0.11957
global softness (*S*)	6.30437	3.93856	4.18165
global electronegativity (χ)	0.16172	0.16359	0.16749

According to Table 7, selected quantum chemical calculations are close to each other in the CAM-B3LYP/6-311G(d,p) and M06-2X/6-311G(d,p) levels. The results in B3LYP/6-311G(d,p) are different from other. However, these results do not represent any errors. When the result of this study is comparable to previous reports,^68^ it is observed that the spherical softness of the studied molecule is quite large at each computational level, and it is lower in the energy gap. In this case, it states that the studied molecule can easily interact.

**Figure 11:**
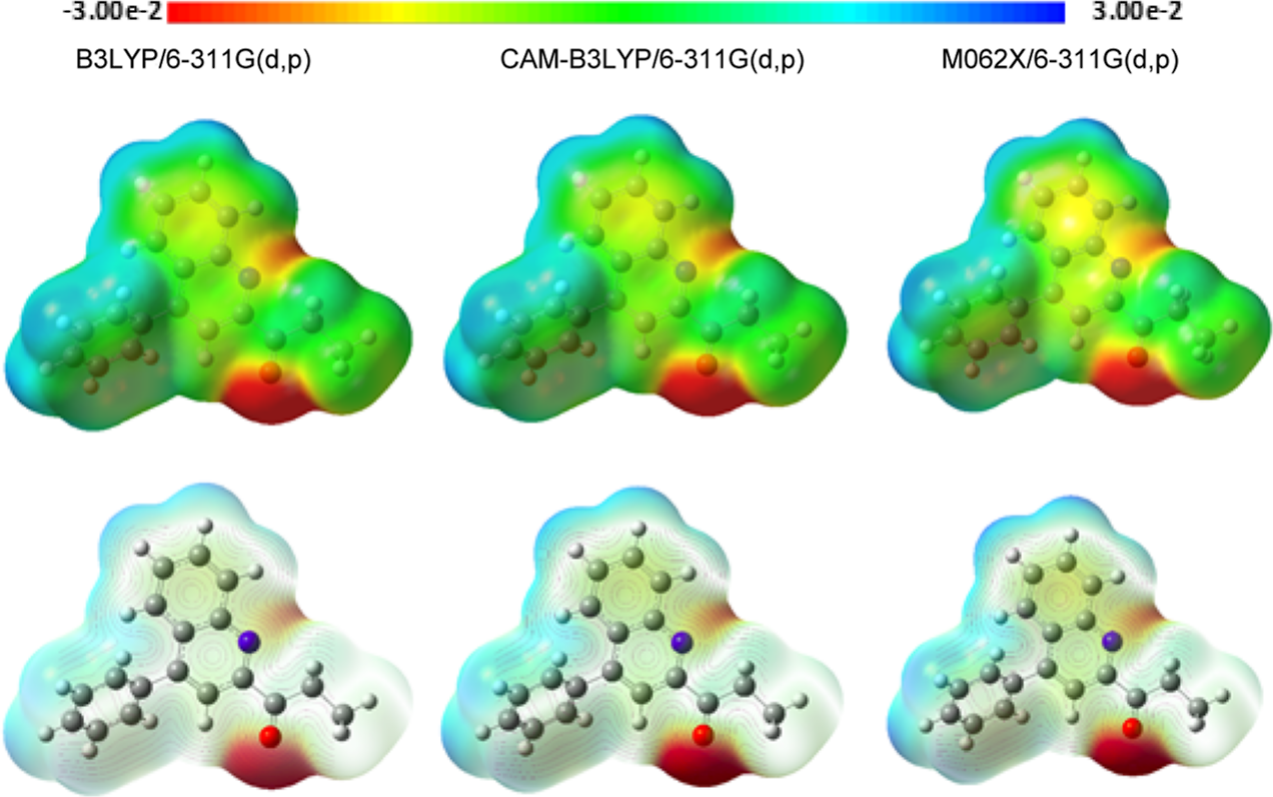
MEP maps and contours of molecule **3** at B3LYP/6-311G(d,p), CAM-B3LYP/6-311G(d,p), and M06-2X/6-311G(d,p) levels in the gas phase.

MEP maps are correlated to electron density on molecular surfaces. These maps can be depicted in nucleophilic or electrophilic active zones. MEP maps and contours of molecule **3** at B3LYP/6-311G(d,p), CAM-B3LYP/6-311G(d,p), and M06-2X/6-311G(d,p) levels in the gas phase are represented in Figure 11. In the MEP contour map, the negative regions (designated as red) of MEP are related to electrophilic attacks, and positive regions (designated as blue) are related to nucleophilic reactivity. According to MEP maps in Figure 11, the environment of the oxygen atom in the carbonyl group with red color acts at the electrophilic reactive site. The surfaces around the nitrogen atom are blue color, which can act as a nucleophilic reactive site.

#### 4.2.8 Thermodynamics Parameters

The thermodynamic parameters of molecule **3** have been calculated to get reliable relations among energetic, structural, and reactivity characteristics of the molecules. Table S3 (Supporting Information) shows the values of thermodynamic parameters such as, Gibbs free energy, entropy allows us to estimate the stability of the compound, and zero-point energy gives information about the nuclear motion of the structure.^69^

#### 4.2.9 NBO Charges

Our interest is to compare the different methods to describe the distribution of electrons in the compound under study and evaluating the sensitivity of the calculated charges to changes in the quantum mechanical method used. The N(1) and the O(1) atoms have the highest negative charges. These charges, being relatively close in space, make this fragment of the molecule a potential coordination site with metal ions. Natural bond orbital (NBO) charges of atoms in studied compound are calculated at each calculation level and given in Table 8.

**Table 8:** NBO Atomic Charges (*e*) of the Molecule **3**

atom	B3LYP	CAM-B3LYP	M06-2X
C1	–0.058	–0.060	–0.064
C2	–0.186	–0.190	–0.192
C3	–0.192	–0.194	–0.196
C4	–0.194	–0.200	–0.201
C5	–0.194	–0.197	–0.199
C6	–0.194	–0.199	–0.201
C7	0.018	0.023	0.018
C8	–0.200	–0.210	–0.212
C9	0.126	0.130	0.131
C10	0.164	0.162	0.163
C11	–0.171	–0.176	–0.177
C12	–0.192	–0.197	–0.199
C13	–0.184	–0.188	–0.190
C14	–0.187	–0.191	–0.194
C15	–0.067	–0.069	–0.073
C16	0.538	0.550	0.550
C17	–0.464	–0.475	–0.481
C18	–0.569	–0.582	–0.583
N1	–0.439	–0.440	–0.433
O1	–0.548	–0.552	–0.549
H2	0.212	0.216	0.219
H3	0.207	0.211	0.213
H4	0.205	0.209	0.212
H5	0.206	0.210	0.213
H6	0.212	0.216	0.219
H8	0.240	0.246	0.248
H11	0.220	0.224	0.227
H12	0.208	0.212	0.214
H13	0.207	0.211	0.213
H14	0.219	0.223	0.226
H17a	0.229	0.233	0.236
H17b	0.229	0.234	0.236
H18a	0.202	0.206	0.207
H18b	0.195	0.199	0.201
H18c	0.201	0.206	0.207

It is interesting to notice that the dipole moment is higher for B3LYP calculation compared with CAM-B3LYP and M06-2X methods. Therefore, B3LYP describes a more polarized structure than the other DFT methods.

#### 4.2.10 Determination of NLO Properties

We carried out the study to calculate the dipolar moment (u) linear polarizability (α) and the first order hyperpolarizability (β) using the B3LYP, CAM-B3LYP, and M06-2X functional with the 6-311G(d,p) basis set in the gas phase. These parameters are important to predict the NLO properties of molecules for their potential application in optoelectronic technologies in the field of telecommunications, signal processing, data storage, microscopy to higher harmonic, etc.^70^ For its part, urea is a reference substance for NLO material for comparative purposes.^71,72^Table 9 shows the dipole moments and static electronic polarizabilities of molecule **3** obtained from the calculated parameters.

**Table 9:** Dipole Moments and Static Electronic Polarizabilities of the Molecule **3** Calculated with B3LYP/6-311G(d,p), CAM-B3LYP/6-311G(d,p), and M06-2X/6-311G(d,p) Levels of Theory

property	urea			molecule **3**		
	B3LYP	CAM-B3LYP	M06-2X	B3LYP	CAM-B3LYP	M06-2X
μ_*x*_	0.0012	0.0014	0.0000	–2.5628	–2.5084	–2.4506
μ_*y*_	–3.6204	–3.6204	–3.6049	2.2797	2.2452	2.2356
μ_*z*_	0.0004	0.0001	0.0000	–0.0981	–0.0957	–0.0983
μ^a^	3.6204	3.6964	3.6049	3.4314	3.3678	3.3186
α_*xx*_	–17.4265	–17.3455	–17.4046	–106.5586	–106.1839	–104.7016
α_*yy*_	–24.9745	–24.978	–25.0079	–114.7163	–114.8909	–113.9694
α_*zz*_	–24.9976	–25.0503	–25.0713	–115.7369	–116.0649	–116.3475
⟨α⟩^b^	–22.2697	–22.2333	–22.2696	–112.3373	–112.3799	–111.6728
(Δα)^b^	8.9713	8.9686	9.1274	15.9477	16.40179	17.2993
β_total_^c^	11.9617	12.3879	11.9143	57.6441	57.3569	55.5248

a In Debye.

b In (×10^–23^) esu.

c In (×10^–30^) esu.

Interestingly, even though both molecule **3** and urea have relatively similar dipole moments, the linear polarizability and hyperpolarizability of molecule **3** are approximately 5 times higher compared to urea. Thus, the large magnitude of ⟨α⟩ and β_total_ indicate that the molecule **3** is highly polarizable; therefore, this molecule **3** could potentially serve as NLO material.

## 5 Conclusions

In the present work, PPA-assisted synthesis of a novel 1-(4-phenylquinolin-2-yl)propan-1-one from 2-aminobenzophenone and pentan-2,3-dione under solvent-free Friedländer quinoline synthesis is shown. The synthesized 1-(4-phenylquinolin-2-yl)propan-1-one (**3**) is confirmed by molecular and structural analysis like FT-IR, ^1^H and ^13^C NMR chemical shifts, and single-crystal XRD techniques. DFT calculations for molecule **3** were measured and calculated using B3LYP/6-311G(d,p), CAM-B3LYP/6-311G(d,p), and M06-2X/6-311G(d,p) basis sets in the gas phase. As a result, it has been found that there is a good correlation between calculated and experimental data. The optimized geometry of the quinoline molecule was compared with the experimental XRD values. DFT calculations of the noncovalent interactions, Hirshfeld surface analysis, NLO properties, thermodynamic properties, and FMO acknowledged chemically active sites of the quinoline compound accountable for its chemical reactivity. The MEP results showed that the most reactive site for any nucleophilic attack is the nitrogen atom, while the most reactive site for any electrophilic attack is the oxygen atom. These findings may provide information about potential reactive regions on the whole molecule. It is interesting to observe that the dipole moment is higher for B3LYP calculation than for CAM-B3LYP and M06-2X methods. Therefore, B3LYP describes a more polarized structure than the other DFT methods.

## Supporting Information Available

General procedure for synthesis, spectral data, and theoretical investigation of molecule **3** (PDF)
The Cambridge Crystallographic Data Centre has deposited CIF files for compound **3** with the CCDC number 2077085. Copies of the data can be obtained, free of charge, on application to CCDC, 12 Union Road, Cambridge, CB2 1EZ, UK. Fax: +44 (0) 1223 336033 or Email: deposit@ccdc.cam.ac.uk (CIF)

## Supplementary Material

ao3c04360_si_001.pdf

ao3c04360_si_002.cif
