# Supplementary material for: Enhanced Method for the Synthesis and Comprehensive Characterization of 1-(4-Phenylquinolin-2-yl)propan-1-one
Source: ACS Omega. 2023 Nov 8;8(46):43573–85. doi: 10.1021/acsomega.3c04360 (PMC10666135; doi:10.1021/acsomega.3c04360)
Supplement: Supplementary file 1 — ao3c04360_si_001.pdf [file ao3c04360_si_001.pdf]

Supporting Information

Enhanced Method for Synthesis and Comprehensive Characterization of 1-(4-phenylquinolin-2-yl)propan-1-one

Satheeshkumar Rajendran,[a,f]*, Rodrigo Montecinos[b], Jonathan Cisterna,[c] Kolandaivel Prabha[d] ,Karnam Jayarampillai Rajendra Prasad[e], Sushesh Srivatsa Palakurthi[f], Alaa A A Aljabali [g], Gowhar A Naikoo[h] , Vijay Mishra[i], Roberto Acevedo[j], Koray Sayin[k], Nitin Bharat Charbe[l*], Murtaza M Tambuwala[m*]

[a] Departamento de Química Orgánica, Facultad de Química y de Farmacia, Pontificia Universidad Católica de Chile, 702843, Santiago de Chile, Chile.

[b] Departamento de Química Física, Facultad de Química y de Farmacia, Pontificia Universidad Católica de Chile, 702843, Santiago de Chile, Chile.

[c] Departamento de Química, Facultad de Ciencias Básicas, Universidad de Antofagasta, avenida Universidad de Antofagasta 02800, Campus Coloso, Antofagasta, Chile

[d] Department of Chemistry, K. S. Rangasamy College of Technology, Tiruchengode- 637215, Tamil Nadu, India

[e] Department of Chemistry, Bharathiar University, Coimbatore- 641046, India

[f] Department of Pharmaceutical Sciences, Irma Lerma Rangel School of Pharmacy, Texas A&M Health Science Center, Texas A&M University, Kingsville, TX 78363, USA.

[g] Department of Pharmaceutical Sciences, Faculty of Pharmacy, Yarmouk University, Irbid 566, Jordan

[h] Department of Mathematics & Sciences, College of Arts & Applied Sciences, Dhofar University, Salalah, PC 211, Oman

[i] School of Pharmaceutical Sciences, Lovely Professional University, Phagwara, Punjab, 144411, India

[j] Facultad de Ingeniería y Tecnología, Universidad San Sebastián, Bellavista 7. Santiago-8420524, Chile.

[k] Department of Chemistry, Faculty of Science, Sivas Cumhuriyet University Sivas 58140, Türkiye.

[l] Center for Pharmacometrics and Systems Pharmacology, Department of Pharmaceutics, College of Pharmacy, University of Florida, Orlando, FL, USA.

[m] Lincoln Medical School, University of Lincoln, Brayford Pool Campus, Lincoln LN6 7TS, UK.

*Corresponding authors: drrsatheeshphd@gmail.com, nitin.charbe@ufl.edu and mtambuwala@lincoln.ac.uk

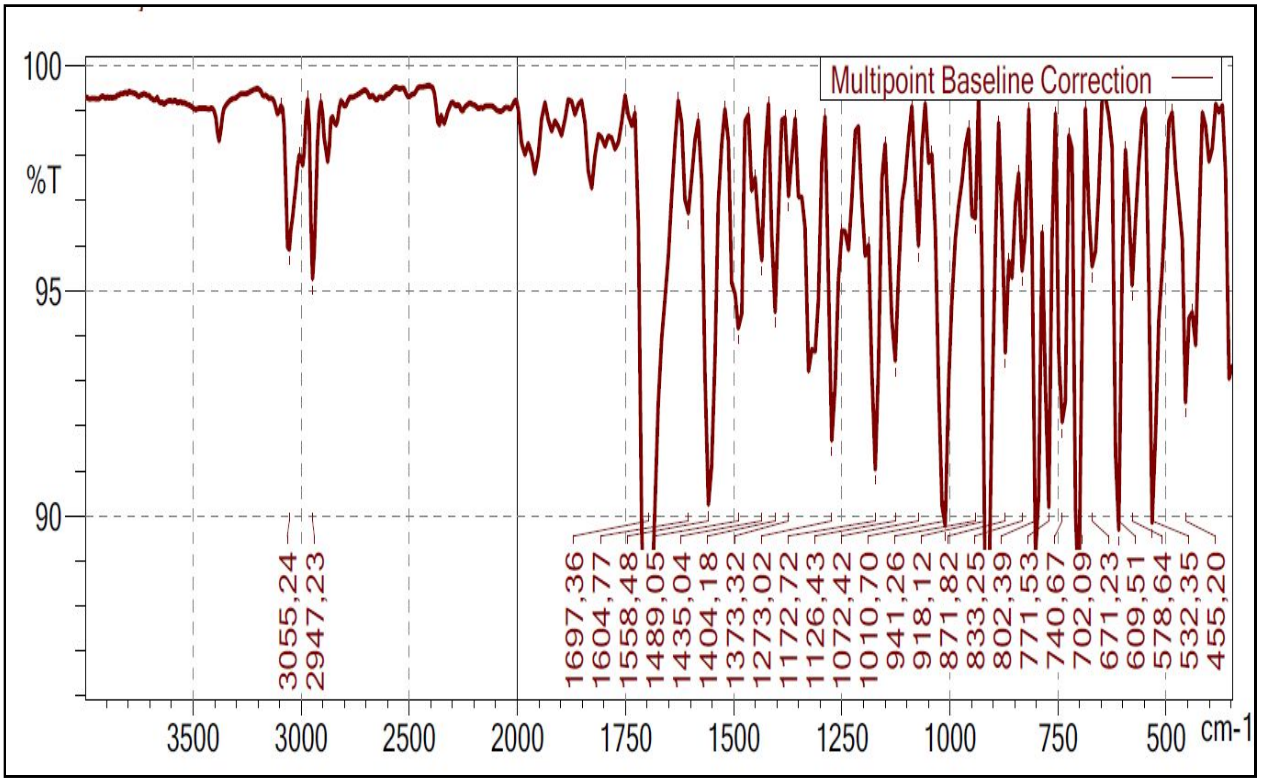

Figure S1 FT-IR spectrum of molecule (**3**)

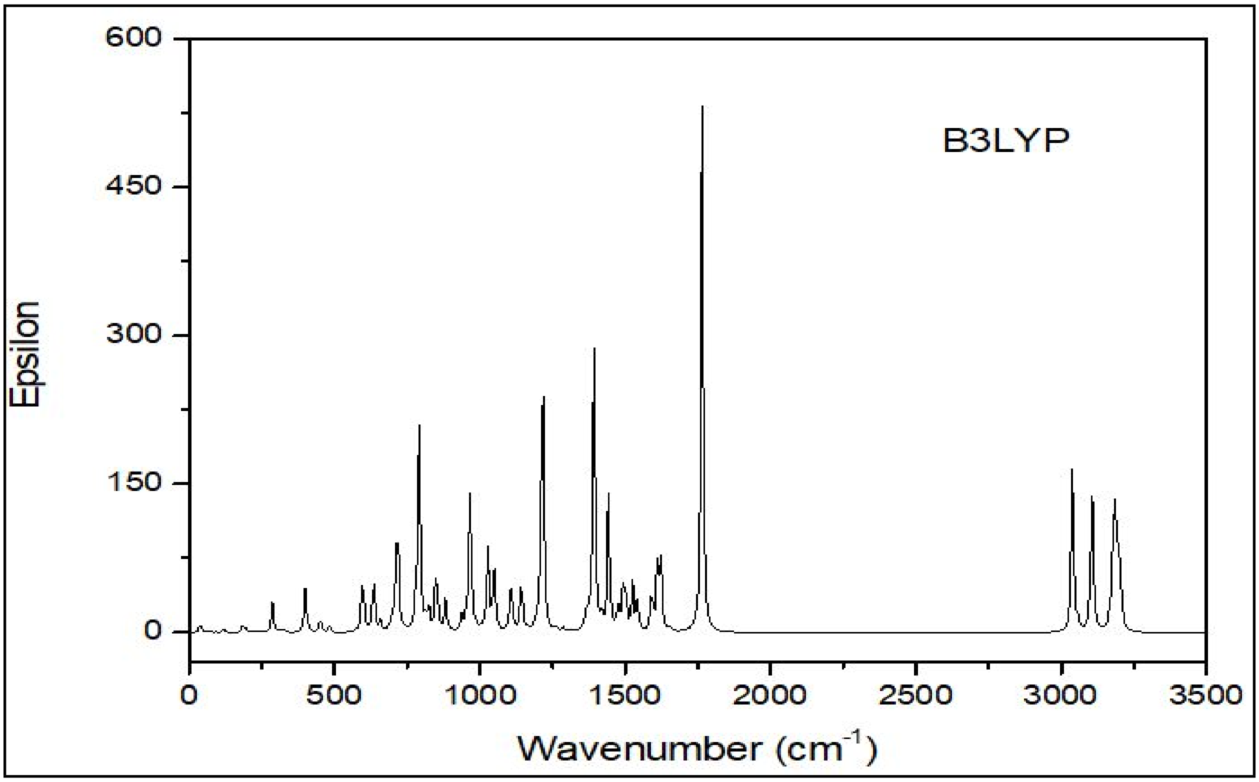

Figure S2 Calculated IR spectrum of molecule (**3**) using B3LYP/6-311G (d,p) levels of theory.

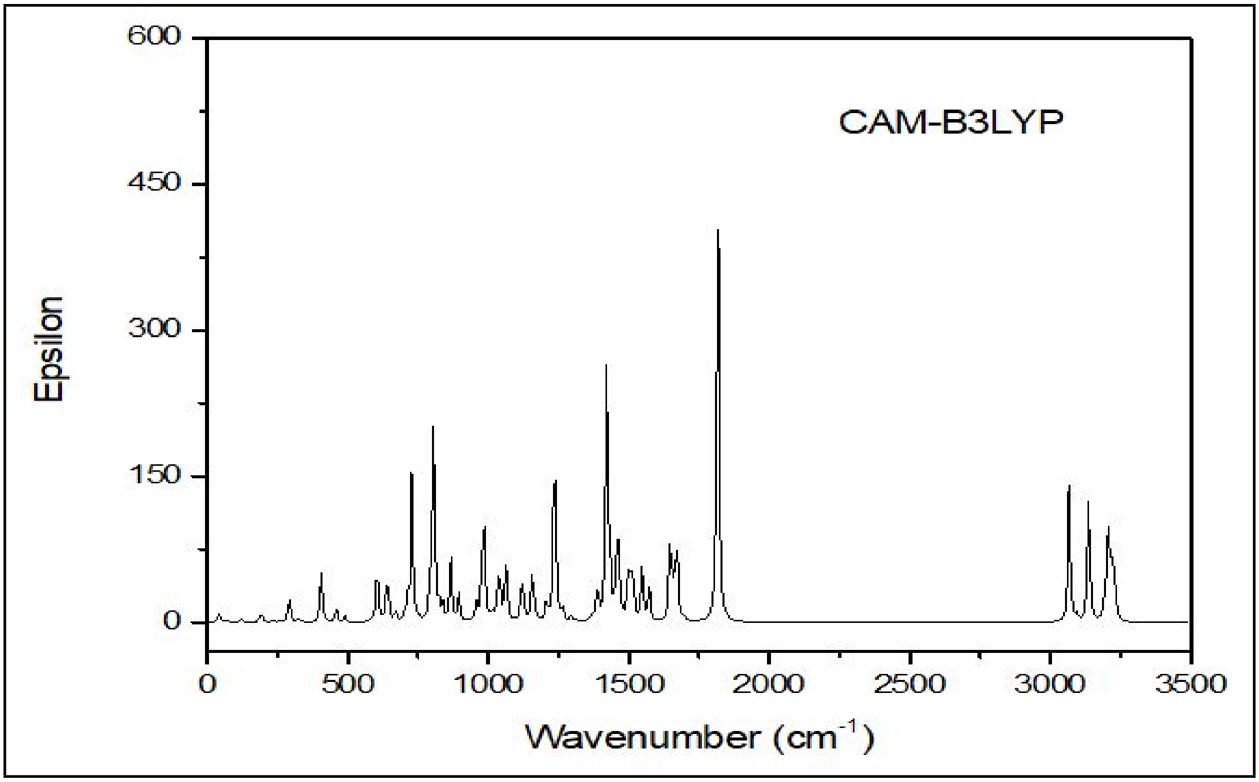

Figure S3 Calculated IR spectrum of molecule (**3**) using CAM-B3LYP/6-311G (d,p) levels of theory.

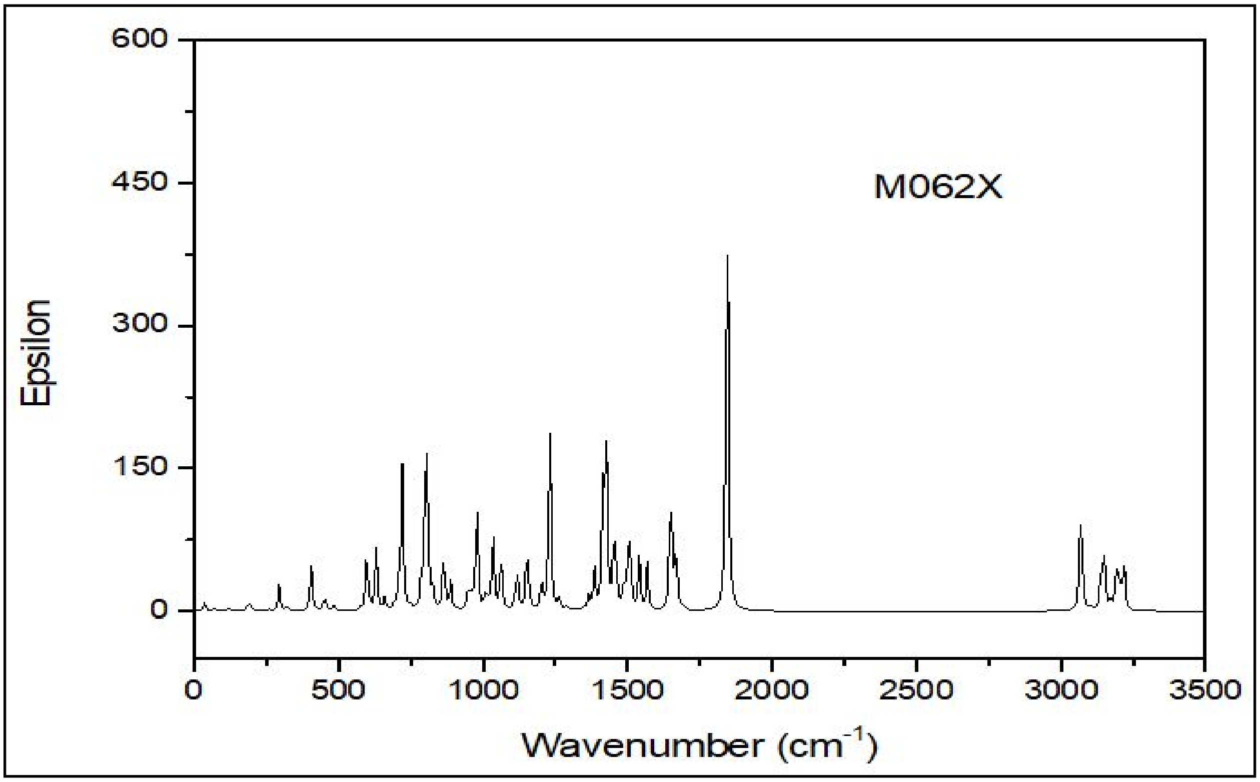

Figure S4 Calculated IR spectrum of molecule (**3**) using M06-2x/6-311G (d,p) levels of theory.

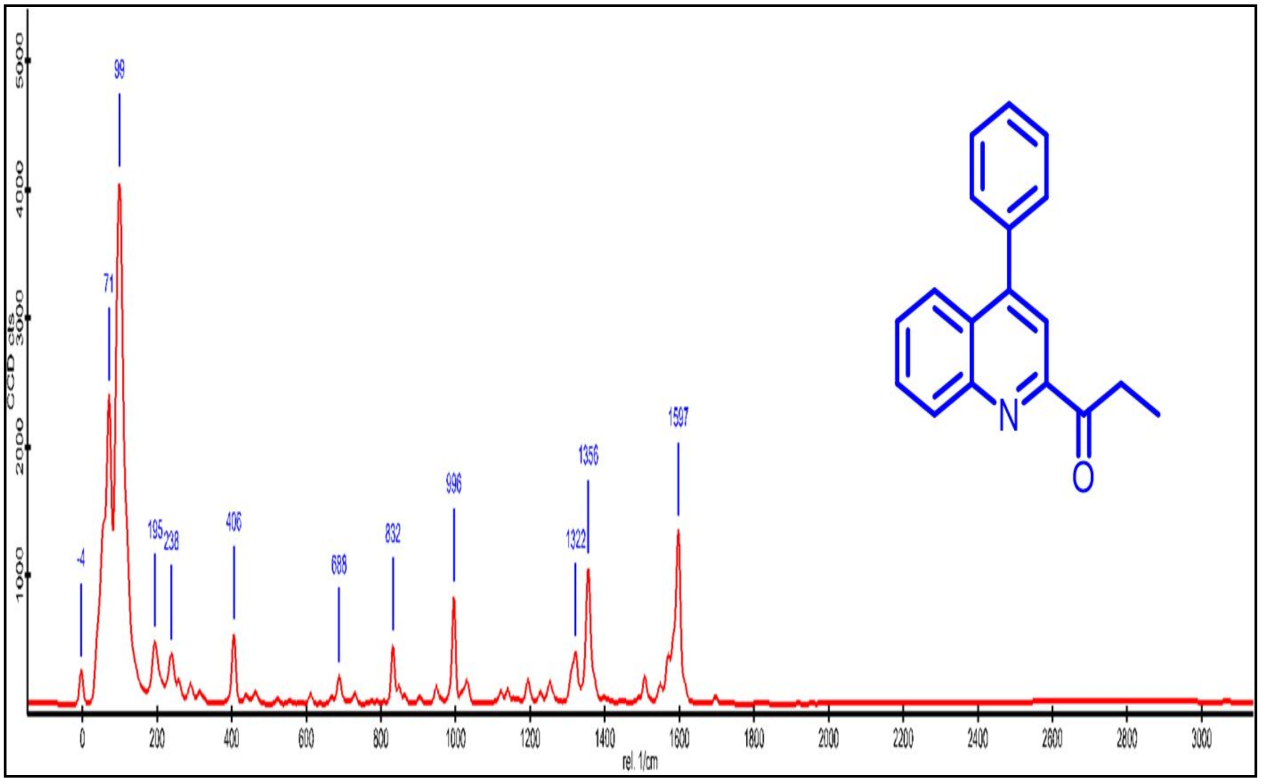

Figure S5 FT-Raman spectrum of molecule (**3**)

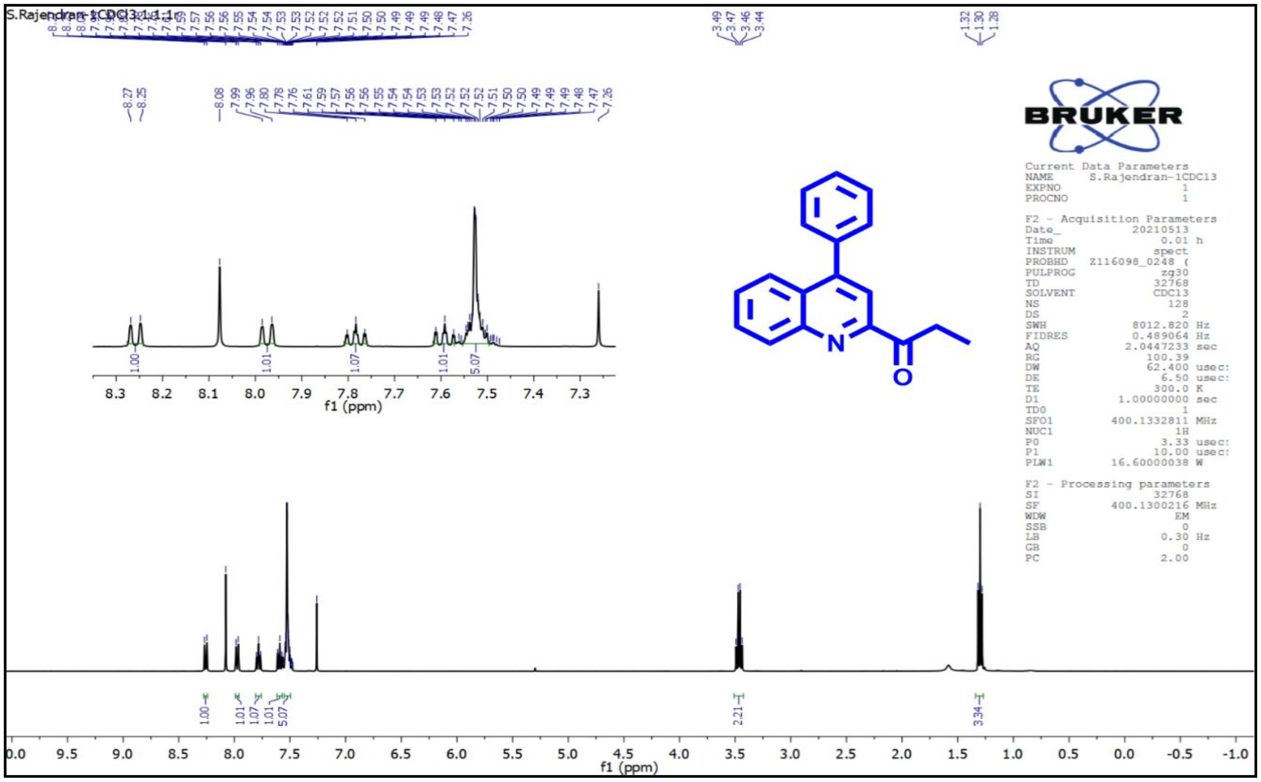

Figure S6 ^{1}H NMR ($CDCl_3$) spectrum of molecule (**3**)

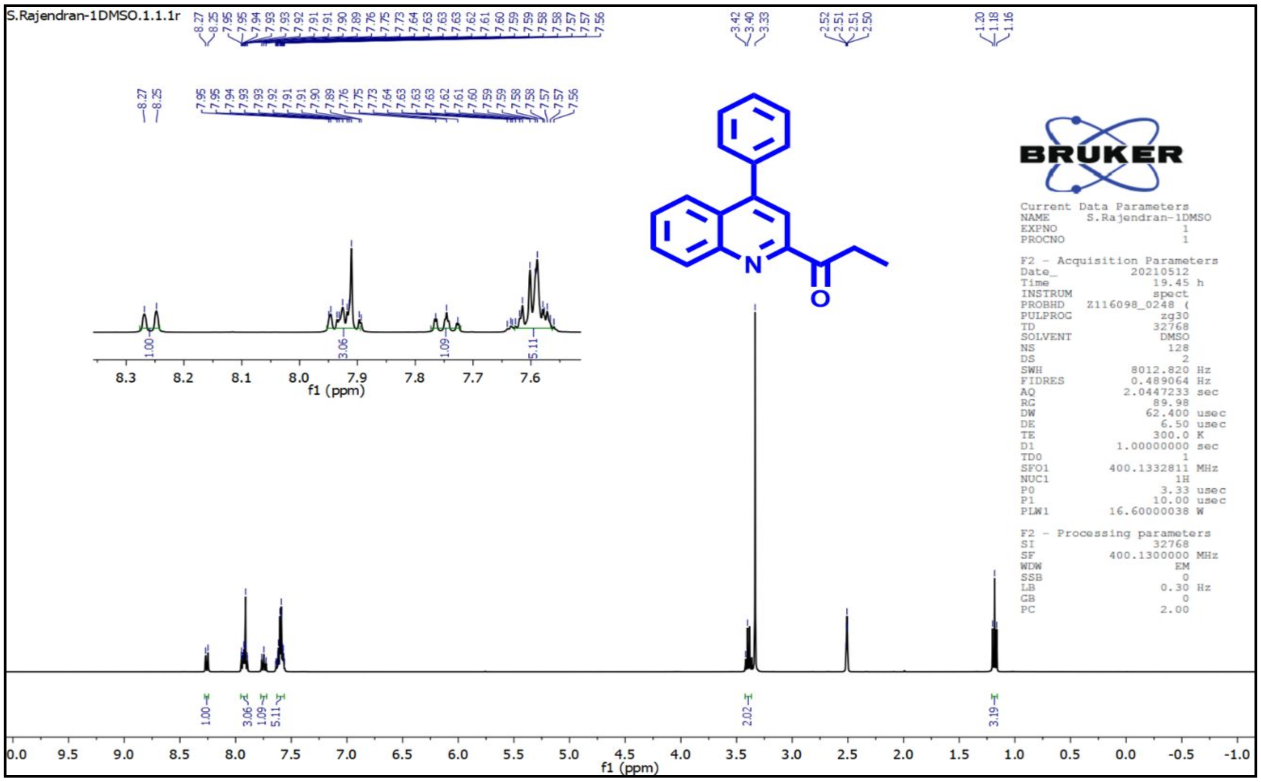

Figure S7 ^{1}H NMR (DMSO-d_6) spectrum of molecule (**3**)

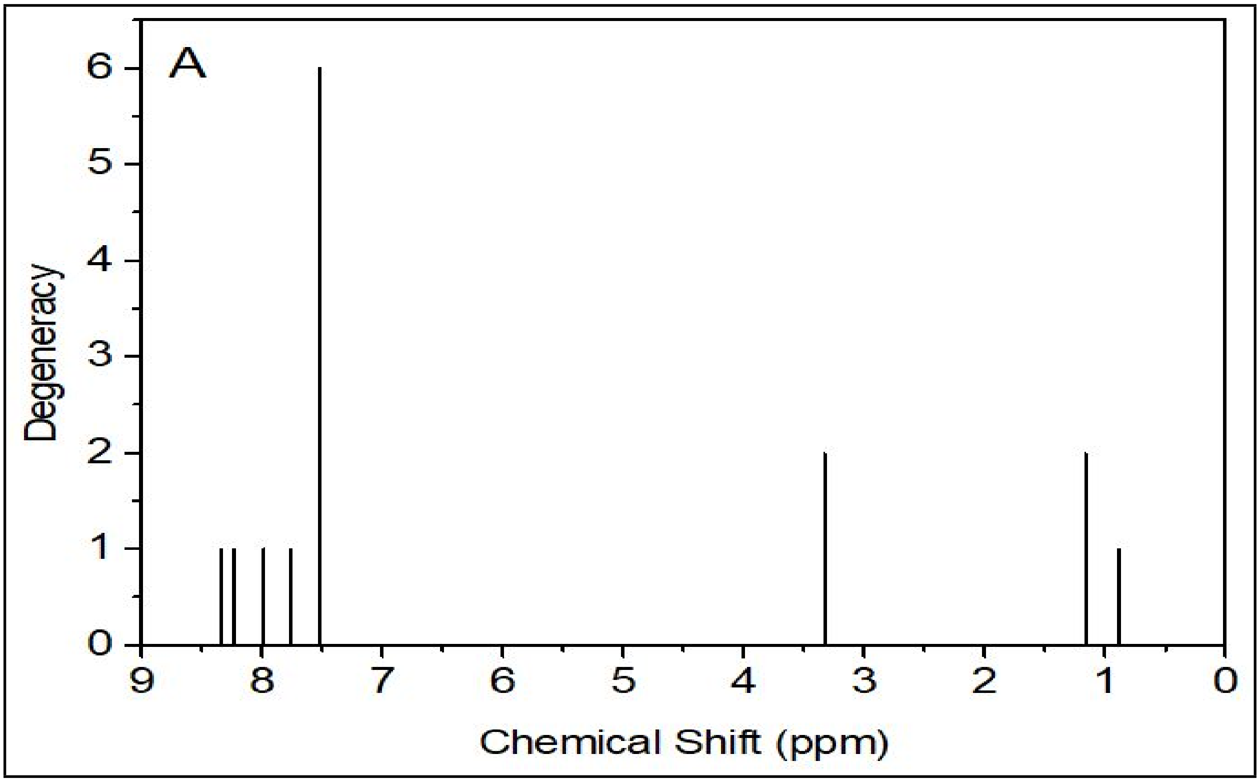

Figure S8 Calculated ^{1}H NMR spectrum of molecule (**3**) using B3LYP/6-311G (d,p) levels of theory.

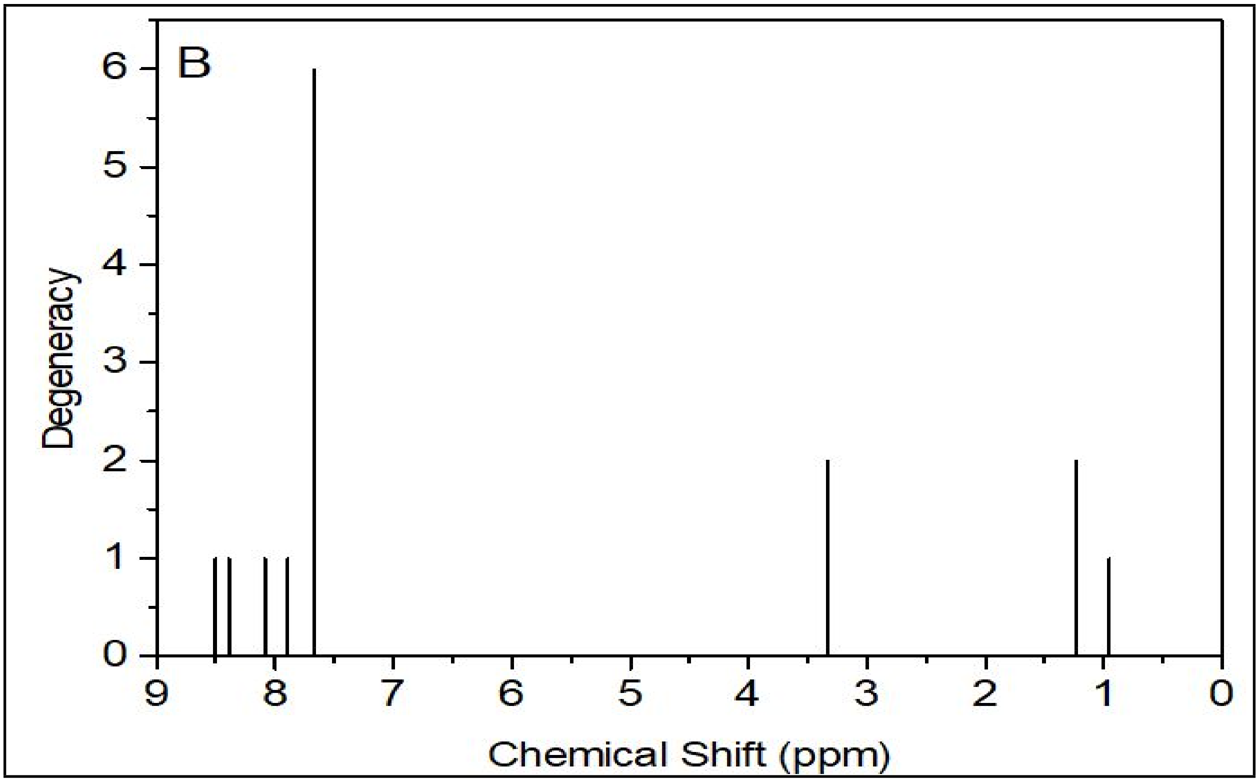

Figure S9 Calculated ^{1}H NMR molecule (**3**) using CAM-B3LYP/6-311G (d,p) levels of theory.

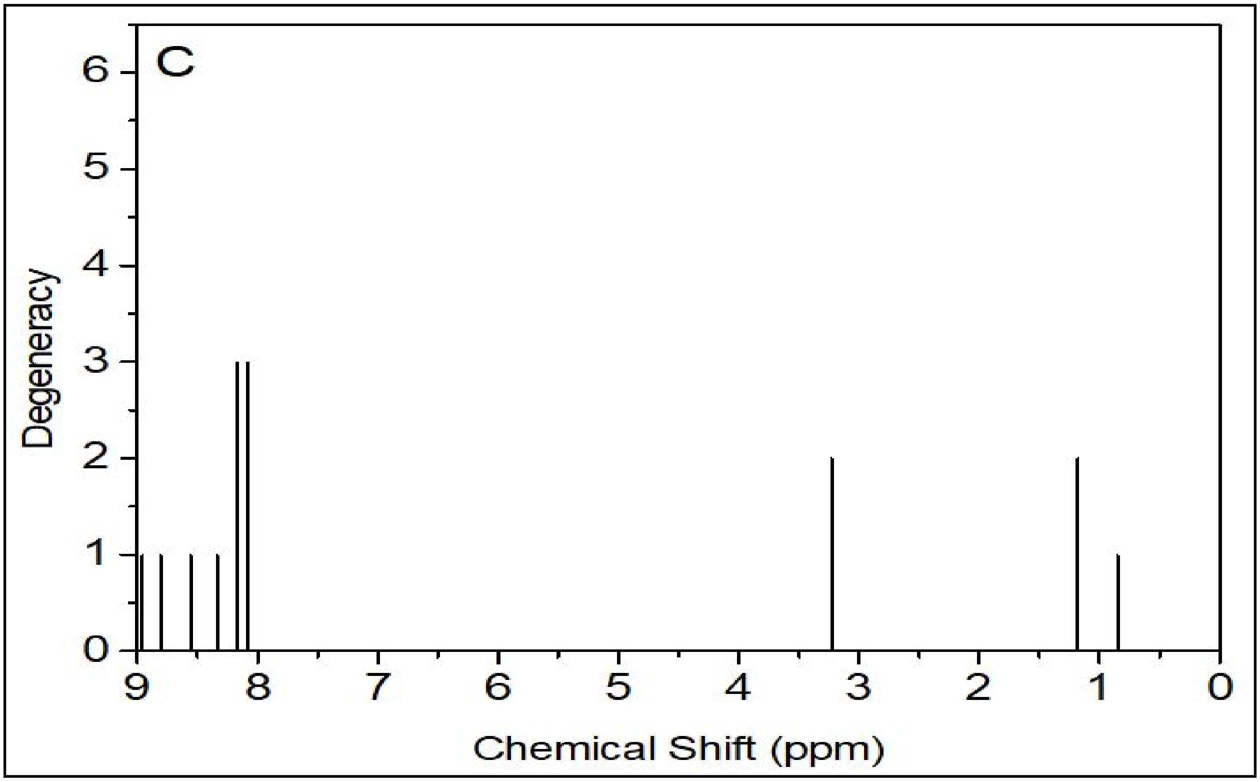

Figure S10 Calculated ^{1}H NMR spectrum of molecule (**3**) using M06-2x/6-311G (d,p) levels of theory.

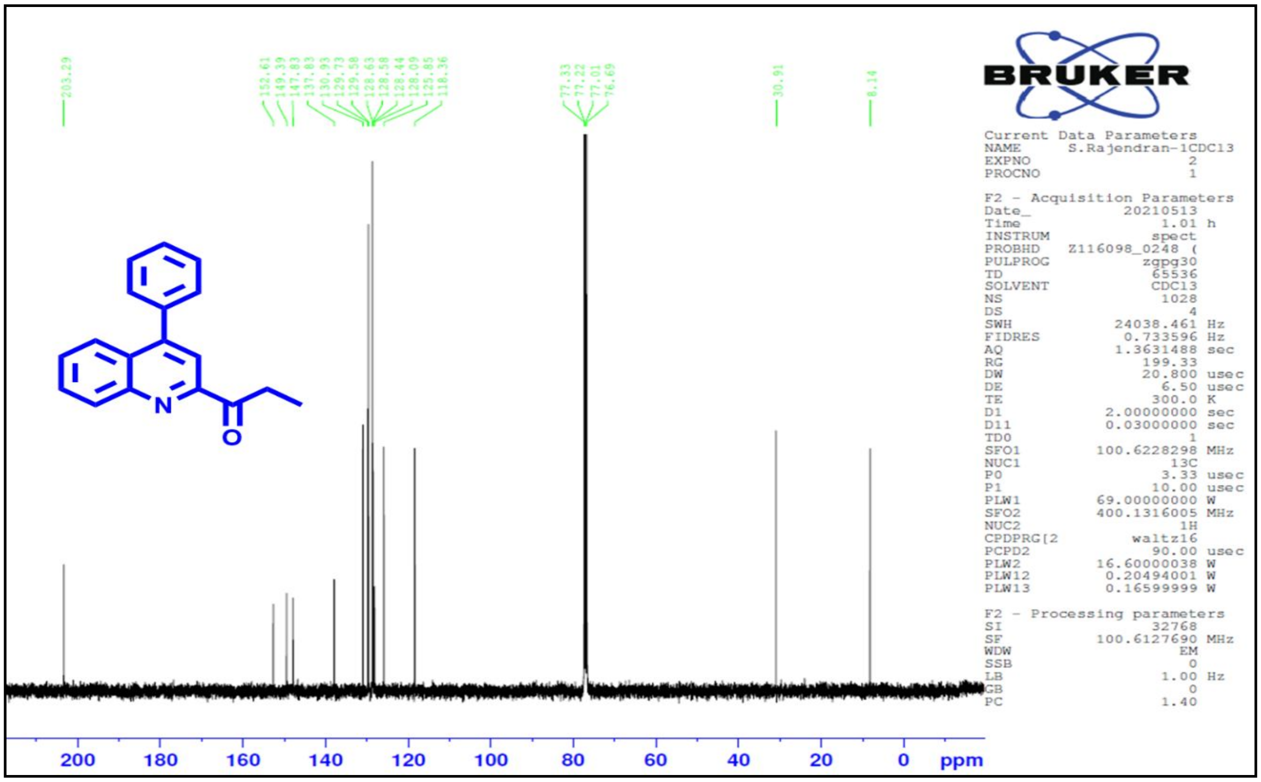

Figure S11 ^{13}C NMR ($CDCl_3$) spectrum of molecule (**3**)

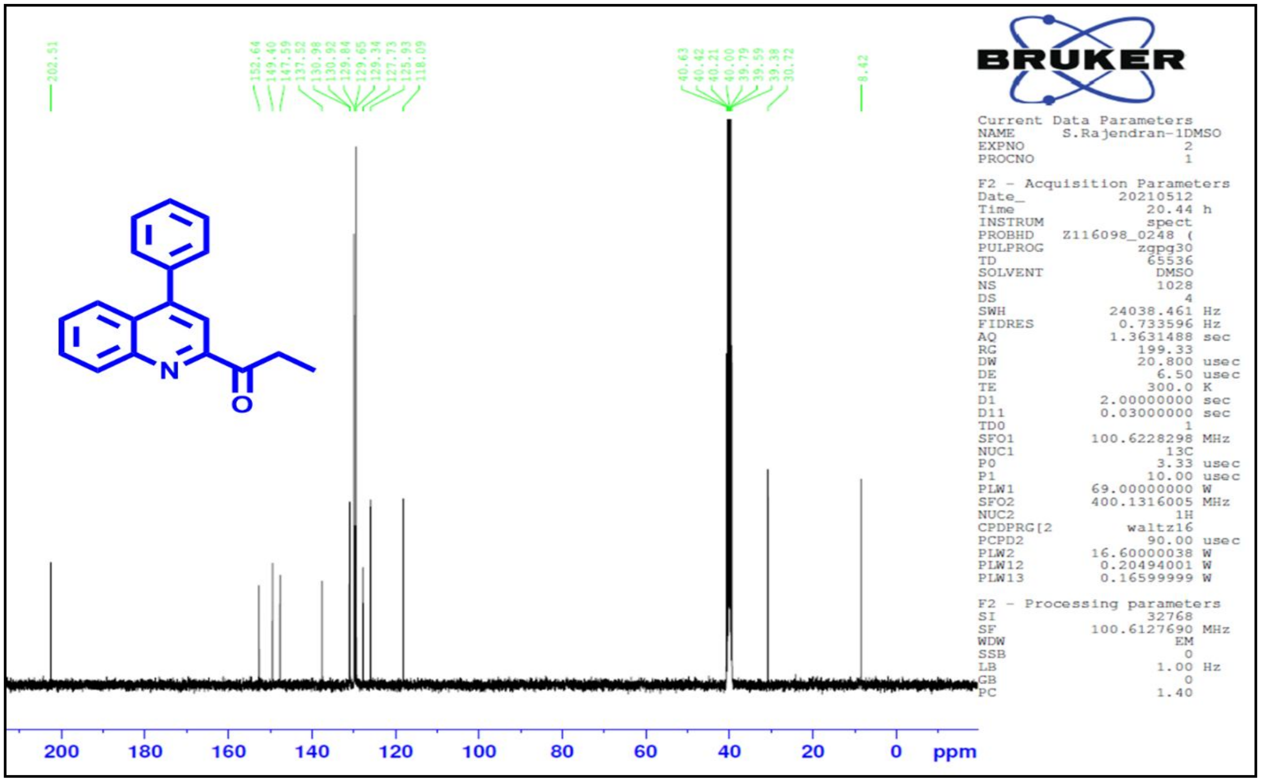

Figure S12 ^{13}C NMR (DMSO-d_6) spectrum of molecule (**3**)

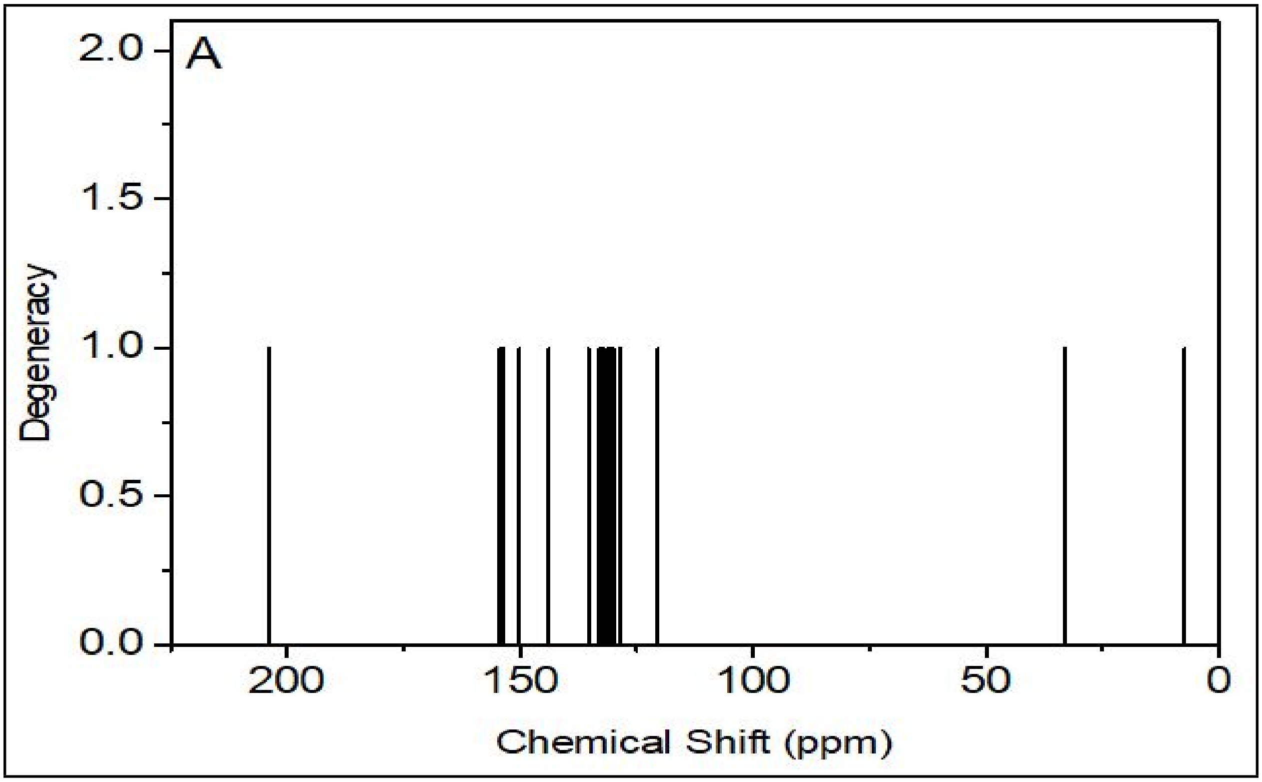

Figure S13 Calculated ^{13}C NMR spectrum of molecule (**3**) using B3LYP/6-311G (d,p) levels of theory.

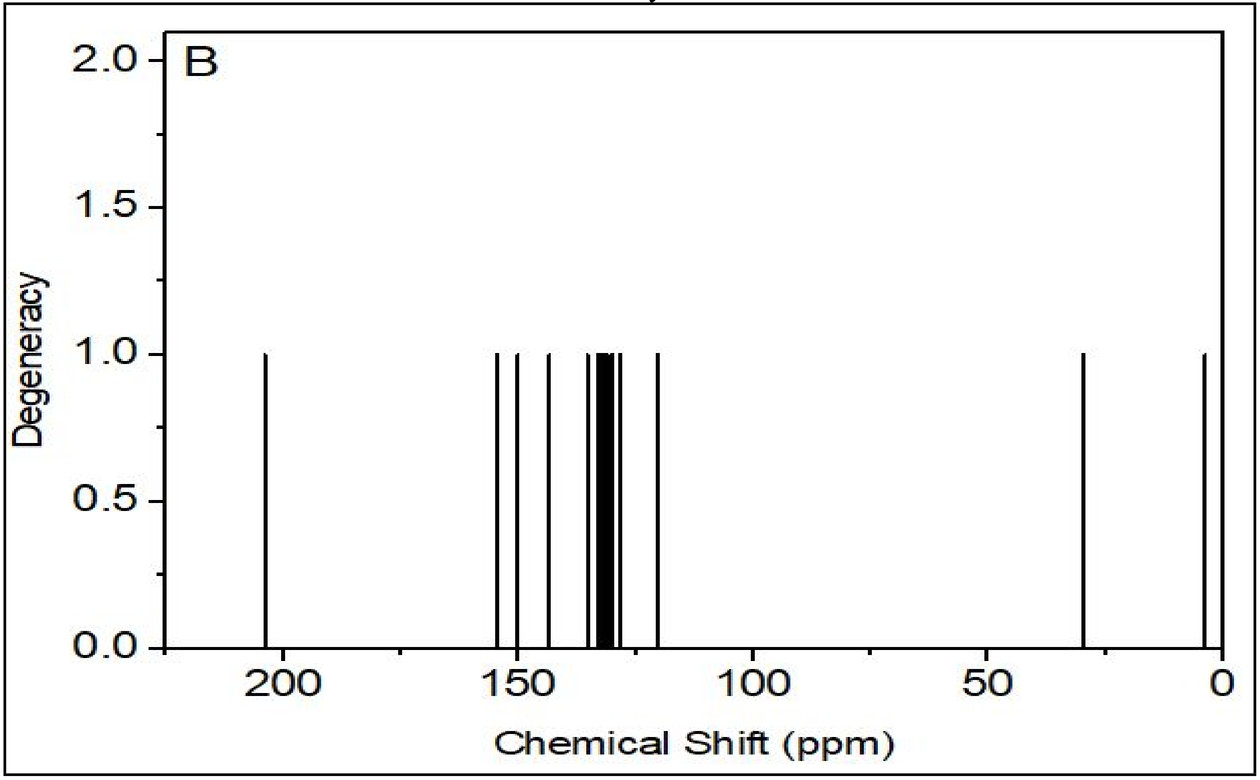

Figure S14 Calculated ^{13}C NMR spectrum of molecule (**3**) using CAM-B3LYP/6-311G (d,p) levels of theory.

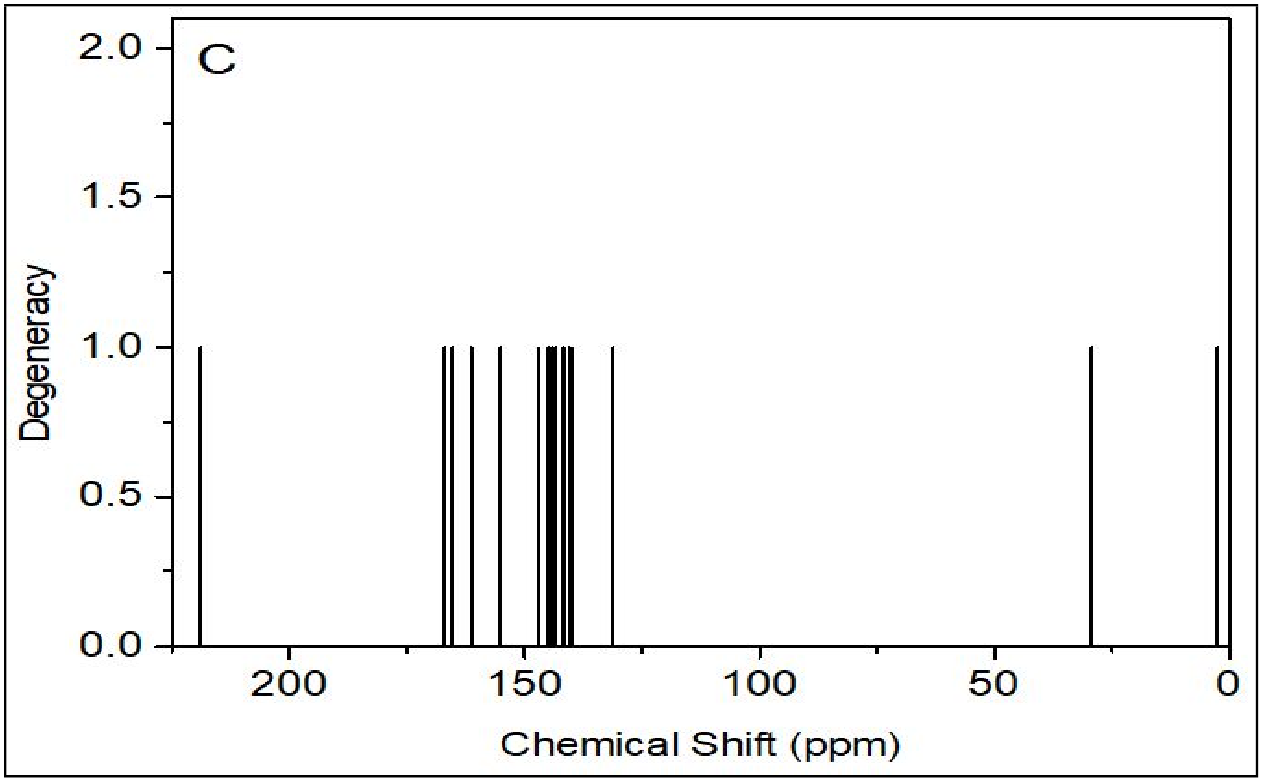

Figure S15 Calculated ^{13}C NMR spectrum of molecule (**3**) using M06-2x/6-311G (d,p) levels of theory.

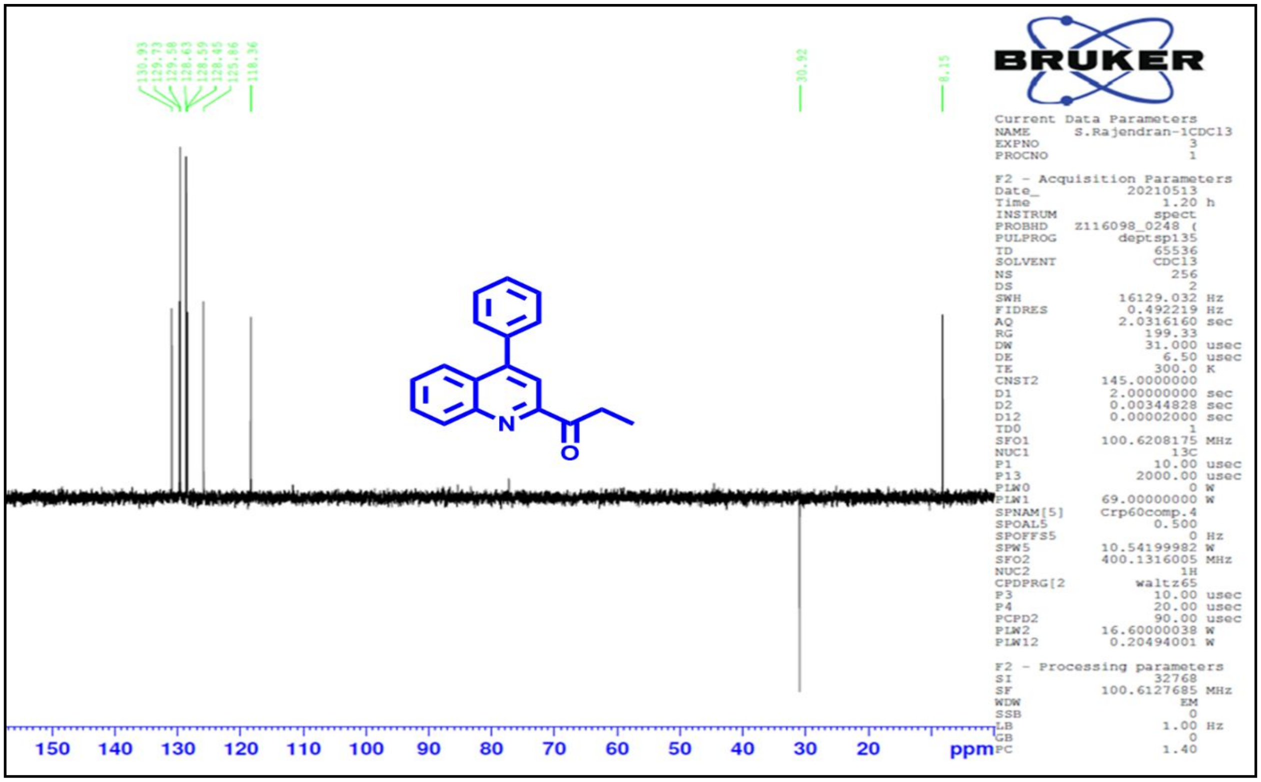

Figure S16 DEPT-135 NMR ($CDCl_3$) spectrum of molecule (**3**)

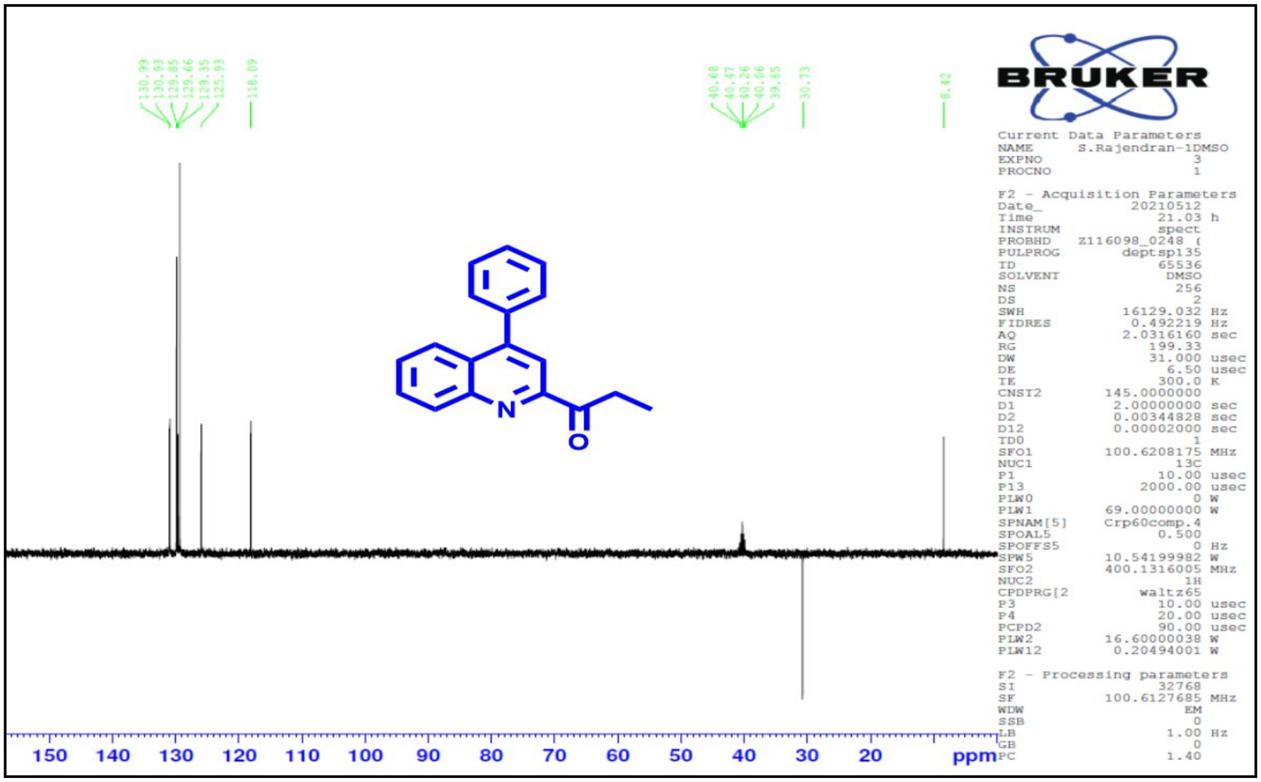

Figure S17 DEPT-135 NMR (DMSO-d_6) spectrum of molecule (**3**)

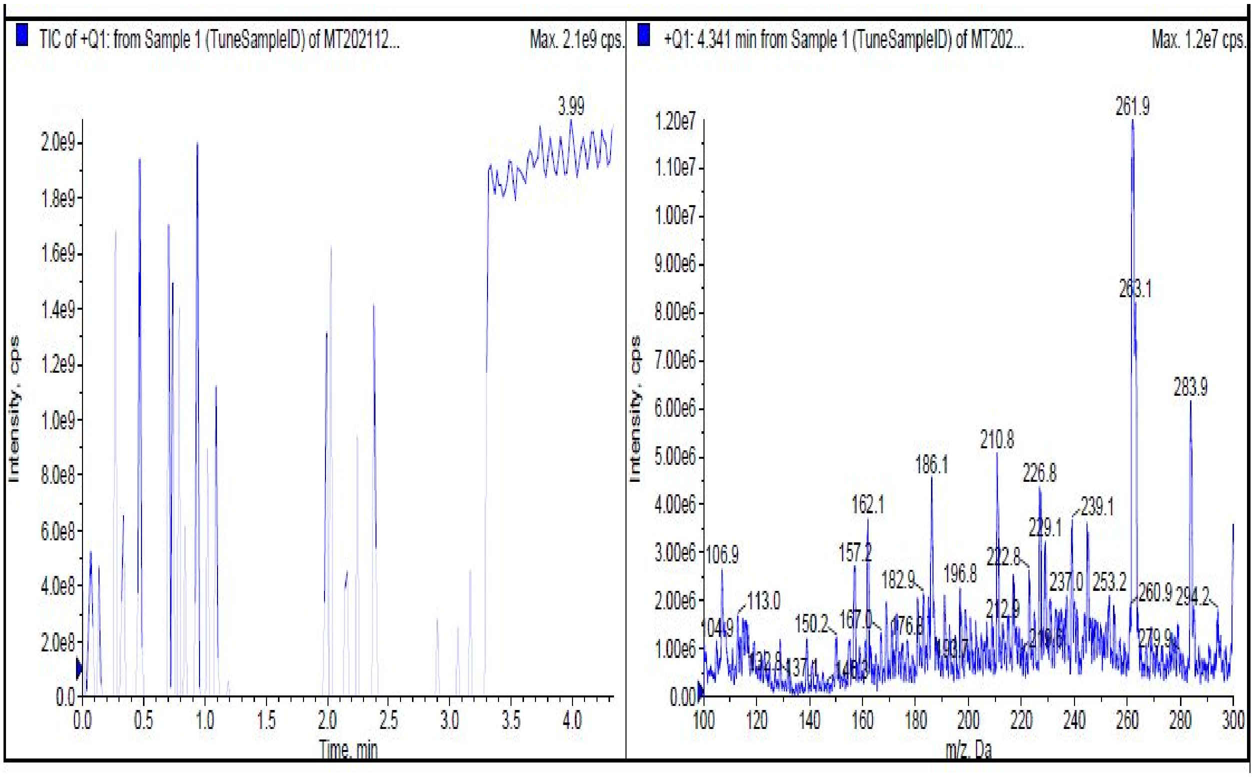

Figure S18 Mass spectrum of molecule (**3**)

Table S1. ^{1}H-NMR Chemical shift δ (ppm)

Atoms	CAM-B3LYP/6-311G	M06-2X/6-311G
C2(H)	7.7	8.15
C3(H)	7.67	8.13
C4(H)	7.69	8.13
C5(H)	7.79	8.25
C6(H)	7.83	8.29
C8(H)	8.46	8.88
C11(H)	8.55	9.01
C12(H)	7.93	8.37
C13(H)	7.73	8.18
C14(H)	8.24	8.7
C17(H2)	3.42	3.32
C18(H3)	1.26	1.2

Table S2. ^{13}C-NMR Chemical Shift δ (ppm)

Atoms	CAM-B3LYP/6-311G	M06-2X/6-311G
C1	160.16	172.15
C2	150.72	162.91
C3	147.09	159.04
C4	148.23	160.85
C5	149.32	161.78
C6	149.65	161.82
C7	171.39	184.04
C8	137.07	148.05
C9	171.95	183.06
C10	167.56	178.97
C11	152.47	164.47
C12	148.48	160.91
C13	147.22	159.1
C14	145.64	157.42
C15	145.64	157.58
C16	220.27	235.15
C17	46.48	46.45
C18	20.29	19.41

	CAM-B3LYP	M06-2X
HOMO-2	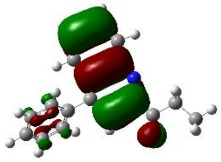<br>-0.31388	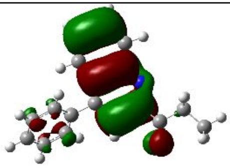<br>-0.31328
HOMO-1	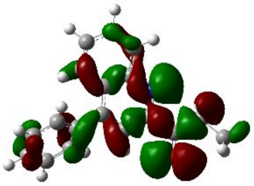<br>-0.31065	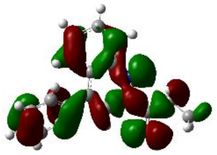<br>-0.31164
HOMO	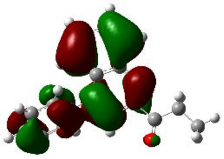<br>-0.29054	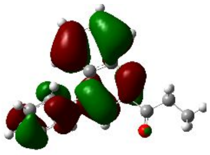<br>-0.28706
LUMO	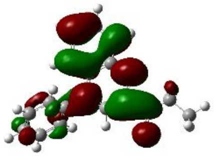<br>-0.03665	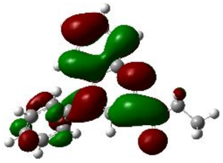<br>-0.04792
LUMO+1	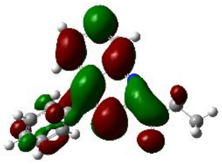<br>0.02345	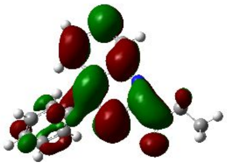<br>-0.01479
LUMO+2	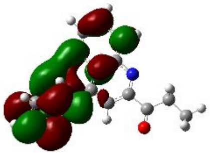<br>0.02822	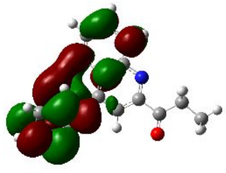<br>0.00965

Figure S19. Molecular orbital energy diagrams (eV) from HOMO-2 to LUMO+2 at CAM-B3LYP/6-311G (d,p) and M06-2X/6-311G (d,p) molecule **3** in gas phase.

Table S3. Thermodynamical parameters of the molecule **3**.

Property	B3LYP	CAM-B3LYP	M062X
Gibbs free energy -standard state (Hartree)	-824.913006	-824.452011	-824.563513
Enthalpy – standard state (Hartree)	-824.849836	-824.391783	-824.501001
S° (cal K^{-1} mol-^{1})	132.953	126.76	131.567
Cv (cal K^{-1} mol^{-1})	64.615	63.596	64.116
Zero-point energy (kcal mol^{-1})	176.31254	178.59096	177.94048
